# Redistribution of garbage codes to underlying causes of death: a systematic analysis on Italy and a comparison with most populous Western European countries based on the Global Burden of Disease Study 2019

**DOI:** 10.1093/eurpub/ckab194

**Published:** 2022-01-21

**Authors:** Lorenzo Monasta, Gianfranco Alicandro, Maja Pasovic, Matthew Cunningham, Benedetta Armocida, Christopher J L Murray, Luca Ronfani, Mohsen Naghavi, GBD 2019 Italy Causes of Death Collaborators, Lorenzo Monasta, Gianfranco Alicandro, Maja Pasovic, Matthew Cunningham, Benedetta Armocida, Luciana Albano, Ettore Beghi, Massimiliano Beghi, Cristina Bosetti, Nicola Luigi Bragazzi, Giulia Carreras, Giulio Castelpietra, Alberico L Catapano, Maria Sofia Cattaruzza, Giulia Collatuzzo, Sara Conti, Giovanni Damiani, Pietro Ferrara, Carla Fornari, Silvano Gallus, Simona Giampaoli, Davide Golinelli, Gaetano Isola, Paolo Lauriola, Carlo La Vecchia, Matilde Leonardi, Francesca Giulia Magnani, Giada Minelli, Marcello Moccia, Paolo Pedersini, Norberto Perico, Alberto Raggi, Giuseppe Remuzzi, Francesco Sanmarchi, Davide Sattin, Brigid Unim, Jorge Hugo Villafañe, Francesco S Violante, Christopher J L Murray, Luca Ronfani, Mohsen Naghavi

**Affiliations:** Institute for Maternal and Child Health IRCCS “Burlo Garofolo”, Trieste, Italy; Department of Pathophysiology and Transplantation, Università degli Studi di Milano, Milan, Italy; Cystic Fibrosis Center, Fondazione IRCCS Ca' Granda Ospedale Maggiore Policlinico, Milan, Italy; Institute for Health Metrics and Evaluation, University of Washington, Seattle, WA, USA; Institute for Health Metrics and Evaluation, University of Washington, Seattle, WA, USA; Institute for Maternal and Child Health IRCCS “Burlo Garofolo”, Trieste, Italy; Institute for Health Metrics and Evaluation, University of Washington, Seattle, WA, USA; Department of Health Metrics Sciences, School of Medicine, University of Washington, Seattle, WA, USA; Institute for Maternal and Child Health IRCCS “Burlo Garofolo”, Trieste, Italy; Institute for Health Metrics and Evaluation, University of Washington, Seattle, WA, USA

## Abstract

**Background:**

The proportion of reported causes of death (CoDs) that are not underlying causes can be relevant even in high-income countries and seriously affect health planning. The Global Burden of Disease (GBD) study identifies these ‘garbage codes’ (GCs) and redistributes them to underlying causes using evidence-based algorithms. Planners relying on vital registration data will find discrepancies with GBD estimates. We analyse these discrepancies, through the analysis of GCs and their redistribution.

**Methods:**

We explored the case of Italy, at national and regional level, and compared it to nine other Western European countries with similar population sizes. We analysed differences between official data and GBD 2019 estimates, for the period 1990–2017 for which we had vital registration data for most select countries.

**Results:**

In Italy, in 2017, 33 000 deaths were attributed to unspecified type of stroke and 15 000 to unspecified type of diabetes, these making a fourth of the overall garbage. Significant heterogeneity exists on the overall proportion of GCs, type (unspecified or impossible underlying causes), and size of specific GCs among regions in Italy, and among the select countries. We found no pattern between level of garbage and relevance of specific GCs. Even locations performing below average show interesting lower levels for certain GCs if compared to better performing countries.

**Conclusions:**

This systematic analysis suggests the heterogeneity in GC levels and causes, paired with a more detailed analysis of local practices, strengths and weaknesses, could be a positive element in a strategy for the reduction of GCs in Italy.

## Introduction

Despite constant improvements in the reporting of causes of death (CoDs), with high-quality CoD data reported via vital registration (VR) systems, a non-negligible share of deaths remains poorly classified. The process of identifying and reporting the correct underlying cause of death (UCoD) can be challenging.^1^

One of the main problems encountered is that of the so-called garbage codes (GCs).^2^ GCs are a set of International Classification of Diseases (ICD) codes that define poorly specified diagnoses not clearly identifying an UCoD. GCs limit the utility of death statistics, undermining their importance as a primary source of information for planning and assessing health policies and interventions.^3^

Common criticalities in UCoD reporting can be traced from certification by physicians to coding.^4^ In high-income countries, GCs are more often related to poor certification. In Italy, coding is done at central level by the National Institute of Statistics (ISTAT). ISTAT applies WHO standards that minimize the number of deaths assigned to ill-defined and trivial causes. Moreover, an ad hoc international software automatically codes about 80% of death certificates to reduce potential errors introduced by coders in manual coding and facilitate international comparisons.^5^ Regarding certification, Italy still relies on paper death certificates. Quality of certification can be affected by (i) lack of information in identifying the UCoD, (ii) lack of importance attributed to death certification or (iii) lack of training of physicians in this specific task. The lack of information should not be relevant in more affluent countries, such as Italy.^6^

Different approaches have been used to classify GCs and reduce their impact, generally involving the redistribution of GC to plausible UCoDs.^1^^,^^7–9^ The Global Burden of Disease (GBD) Study tries to generate comparable cause-specific mortality estimates from a collection of imperfect, heterogeneous data,^10–12^ by redistributing GCs to UCoDs. Accounting for deaths assigned to GCs is one of the key data processing steps in creating comparable cause-specific mortality estimates, by time, age group, sex and location.

The issue of GCs was addressed for Italy by the Italian GBD Initiative, a network of Italian GBD collaborators now comprising more than 100 collaborators from over 25 research institutions, including the National Institute of Health (*Istituto Superiore di Sanità*). During the last round of estimates’ revisions, several discrepancies were found between official data and GBD estimates. These discrepancies might induce scepticism when users of CoD statistics need to bring together official VR data and GBD estimates. Most of them were the consequence of a substantial difference in the scope of official data vs. GBD estimates, which translates in GBD into the redistribution of CoDs—identified as GCs by the GBD—to UCoD.^2^^,^^10^^,^^13^

The objective of the present paper is to describe the GCs identified by the GBD 2019 study, and the effects of their redistribution on UCoD estimates for Italy, and to discuss how GBD estimates finally reconcile with the official statistics. Using estimates from the GBD 2019 cycle, we describe temporal changes (1990–2017), and make comparisons between Italy and other Western European countries with populations above 10 million inhabitants, as well as among the 19 Italian Regions and two Autonomous Provinces.

It is not within the scope of this paper to describe national reporting systems and solutions adopted to reduce the burden of GCs. However, the proposed approach can help solve the misunderstanding around the differences between VR data and GBD estimates, can lead to similar analyses in other Western European countries, and provide useful information to build up a common platform for discussion and intervention.

## Methods

### Overview on GC definitions and redistribution methods

The univocal assignment of ICD codes to GBD CoDs is mapped and constantly updated. Details are available elsewhere.^10^^,^^14^ According to the GBD Study, reported CoDs that do not identify specific UCoDs are identified as GCs, and can be classified in two main categories:

- CoDs that cannot be considered UCoDs, either because they are intermediate (e.g. sepsis or heart failure) or immediate causes (e.g. cardiac arrest or respiratory failure).^2^
- Generic causes that do not identify a specific UCoD, e.g. unspecified type of diabetes or cancers of unknown primary (CUP).

GCs are classified into four classes,^13^^,^^15^ according to their importance in terms of policy implications, and to the Levels of the GBD cause list across which they can be redistributed:

- Class 1 comprises GCs that could be redistributed to any UCoD in any of the three Level 1 cause groups of diseases and injuries (e.g. sepsis): Communicable diseases, non-communicable diseases and Injuries. They are the most difficult to redistribute and require the most modelling.
- Class 2 comprises GCs that could be redistributed to any UCoD in one or two of the three Level 1 cause groups of diseases and injuries (e.g. unspecified and undetermined intent of injury).
- Class 3 includes GCs for which the UCoD is likely part of the same ICD chapter (e.g. CUP).
- Class 4 is the one with less policy implications and identifies GCs for which the UCoD is likely to be attributed to a single disease or injury (e.g. unspecified type of stroke).

Details on the methods and algorithms developed for redistribution of GCs to UCoDs are described elsewhere.^13^

### Overview of present analysis

The specific analysis we conducted was meant to identify the main GCs requiring redistribution to UCoDs, and how they got redistributed. We also analysed from which GCs the UCoDs most affected by redistribution received additional deaths. We conducted our analysis based on the GBD 2019 cycle estimates, and focused on the years for which we had both official CoD data for Italy (from the ISTAT) and GBD estimates, ranging from 1990 to 2017.

The GBD 2019 study complies with the Guidelines for Accurate and Transparent Health Estimates Reporting (GATHER) statement.^16^

The analysis was done on both sexes combined and all ages together. Depending on the analyses and comparisons, we chose to use all age or age-standardized rates. In a mostly homogeneous context such as Western Europe, we decided to compare Italy with similar countries, considering the complexity of a national health system greatly depends on the size of a country’s population. We thus conducted comparisons between Italy and Western European countries with more than 10 million inhabitants, namely Belgium, France, Germany, Greece, the Netherlands, Portugal, Spain, Sweden and the UK. For some countries (France, Belgium and Greece), we could only reach 2016, due to the unavailability of country VR data for the year 2017. From 1996 (the Netherlands) to 2014 (Greece), all countries shifted from the ICD9 to the ICD10 coding system. In addition, ICD9 CoD data for some countries were only available in the aggregated Basic Tabulation List form (ICD9 BTL): Belgium (ICD10 in 1998, in BTL 1990-1991), France (ICD10 in 2000), Germany (ICD10 in 1998, in BTL 1990-1994), Greece (ICD10 in 2014, in BTL 2007, 2009-2013), the Netherlands (ICD10 in 1996), Italy (ICD10 in 2003), Portugal (ICD10 in 2002), Spain (ICD10 in 1999), Sweden (ICD10 in 1997), the UK (Scotland: ICD10 in 2001; Northern Ireland, England and Wales: ICD10 in 2002).

We also compared the 19 Italian Regions (Piemonte, Valle d’Aosta, Lombardia, Veneto, Friuli-Venezia Giulia, Liguria, Emilia-Romagna, Toscana, Umbria, Marche, Lazio, Abruzzo, Molise, Campania, Puglia, Basilicata, Calabria, Sicilia and Sardegna) and the two Autonomous Provinces of Bolzano and Trento.

For Western European countries and subnational locations for Italy, we compared the rate of GCs over the total number of reported deaths, age-standardized, for all the GCs, and separately for Classes 1 and 2, and Classes 3 and 4. For the same countries and subnational locations for Italy, we then ranked the first 10 GCs for the last available year (2017 or 2016) by percentage of attributed deaths over total number of deaths. The rationale was to understand if differences in VR systems could lead to particular patterns in the relative weight of GCs. Differences could also partly be the reflection of different epidemiological profiles of countries and locations.

To be able to study the evolution of the ranking of main GCs for Italy, we selected the first 15 for the years 1990 and 2017, altogether covering more than 90% of all GCs for the respective years. The same analysis was conducted on age-standardized GC rates per 100k population.

For Italy, and for each subnational location, we analysed the first 15 UCoDs affected by redistribution. These first 15 causes, with the exception of the Province of Trento, Marche and Sardegna, accounted for over 90% of the overall causes requiring redistribution.

At the national level for Italy, the year 2017, we selected the first 15 GCs in terms of the number of deaths, and showed to which UCoDs these got redistributed.

Finally, for Italy, the year 2017, we analysed how the first 15 Level 4 UCoDs were affected by redistribution, by reporting which GCs mainly received additional deaths. For each UCoD, we show the first 10 GCs, always covering more than 90% of the redistribution.

## Results

In 1990, in Italy, the age-standardized percentage of GCs over total deaths was 31% (table 1). Sweden had the lowest percentage (23%), while Greece and Portugal had the highest (47 and 51%). Supplementary table S1 shows the crude all-age percentage of GCs over total deaths, with differences from table 1 attributable to the different age structure of the countries considered.

**Table 1:** Percentage of ‘garbage codes’ over all CODs reported, age-standardized, both sexes

Countries	1990	1995	2000	2005	2010	2011	2012	2013	2014	2015	2016	2017
UK	25.30	26.32	27.29	25.88	24.46	23.07	22.61	22.65	21.70	22.29	21.80	22.06
Sweden	22.91	23.13	23.09	22.46	25.53	25.44	24.88	23.86	23.31	24.85	23.56	22.92
Spain	38.53	33.61	30.40	29.72	26.26	26.59	26.87	25.42	25.28	25.70	24.05	24.31
Germany	30.20	28.83	28.93	27.21	26.02	24.72	24.70	24.79	24.50	24.92	24.75	24.78
**Italy**	31.23	30.90	30.23	29.35	27.62	26.38	26.61	26.51	26.60	27.42	25.58	25.82
The Netherlands	30.15	30.40	31.16	29.22	27.00	26.39	26.17	27.01	26.31	26.37	26.28	25.90
Belgium	32.03	32.33	30.15	29.65	31.17	30.82	31.59	30.82	30.66	31.04	30.80	
France	36.64	35.66	33.91	32.41	32.01	31.54	32.12	32.45	31.29	31.86	32.06	
Portugal	51.35	50.45	49.80	48.85	41.81	40.20	40.81	37.86	32.49	32.66	32.78	31.06
Greece	46.98	45.77	45.01	42.68	37.26	36.80	36.21	33.94	34.87	33.90	33.15	

Comparison among most Western European countries with more than 10 million inhabitants.

From 1990 to 2017, the percentage of GCs decreased for Italy as well as for the other countries considered, with the exception of Sweden which, however, had the lowest level in 1990 and second lowest in 2017. In Portugal and Greece, the percentage of GCs dramatically declined, but still remained above 30%.

Considering the repartition of GCs by Class of attribution, for Italy and most countries, the decrease was mostly due to Classes 3 and 4 GCs (Supplementary tables S2 and S3). Looking at Classes 3 and 4, all countries had more than 25% reductions, with the exception of Belgium which had a 4.5% increase. Classes 1 and 2, instead, showed increases for five countries (Sweden, the UK, Italy, the Netherlands and France), but also marked decreases (above 30%) for Portugal, Greece and Spain. However, the repartition of GCs into Classes 1 and 2 or Classes 3 and 4 does not seem to be associated with the overall percentage of GCs over the total (Supplementary figure S1).

The analysis of the main GCs in terms of percentage over total reported deaths shows heterogeneity in the comparison among the Western European countries considered (Supplementary table S4). Unspecified type of stroke is the first GC requiring redistribution for Italy, representing 5% of all CoDs. Percentages vary among countries, ranging from 2.5% in Spain to 8.4% in Greece. Unspecified type of diabetes is the second most frequent GC for Italy (2.3%), the highest value among the countries considered, followed by unspecified heart diseases, unspecified lower respiratory infections, exposure to unspecified factor (ICD code: X59), sepsis, CUP, each of these accounting for more than 1% of total deaths.

Similar to what we did for the select Western European countries, we carried out a comparison among Italian subnational locations in terms of rate of GCs over total deaths, age-standardized (table 2). We looked at the time trend 1990–2017. This analysis was conducted for all GCs, and separately for Classes 1 and 2, and Classes 3 and 4 (Supplementary tables S5 and S6). We noticed some heterogeneity among Italian locations, in the percentage of age-standardized GCs. We also noticed an overall improvement from 1990 to 2017, again, more evident in Classes 3 and 4. For all GCs (table 2), the best performing location in 2017 was Bolzano, followed by Valle D’Aosta, Sardegna and Marche, while the Regions with the highest rates of GCs were Campania, Calabria and Sicilia.

**Table 2:** Percentage of ‘garbage codes’ over all CODs reported, age-standardized

Sub-national location name	1990	1995	2000	2005	2010	2011	2012	2013	2014	2015	2016	2017
Piemonte	31.05	30.62	29.28	27.93	26.65	25.06	25.53	25.61	25.40	26.40	23.23	24.40
Valle d'Aosta	30.86	28.86	26.34	23.27	27.24	21.45	18.93	26.40	21.16	25.81	19.18	21.83
Lombardia	29.35	28.64	27.36	26.82	24.89	24.29	23.91	24.23	24.21	25.20	23.86	23.37
Provincia autonoma di Bolzano	28.85	26.42	26.29	20.89	20.26	17.53	20.55	22.21	21.92	20.66	18.93	20.49
Provincia autonoma di Trento	26.35	27.92	23.11	25.50	19.13	18.67	20.68	25.15	26.27	25.17	22.86	23.56
Veneto	27.11	27.08	28.16	27.69	25.10	23.81	24.80	25.18	24.36	24.75	23.53	23.67
Friuli-Venezia Giulia	29.67	28.42	25.97	26.90	24.86	24.05	24.59	23.95	24.14	26.17	23.34	23.06
Liguria	31.60	34.66	33.16	32.91	29.26	26.92	27.78	26.20	27.70	28.04	28.09	27.73
Emilia-Romagna	28.45	28.29	27.93	26.37	24.77	22.43	23.71	24.03	24.39	24.36	23.47	23.38
Toscana	32.59	31.69	30.30	29.23	27.97	26.10	27.43	26.46	26.44	26.57	25.74	24.96
Umbria	30.12	28.25	27.95	27.06	24.85	23.99	24.18	26.48	24.93	26.25	26.04	23.53
Marche	29.57	28.39	28.54	28.47	27.46	24.63	24.60	23.13	24.15	25.37	21.97	22.11
Lazio	27.99	29.85	29.85	28.48	27.40	26.06	25.70	25.55	26.35	27.47	24.43	25.40
Abruzzo	32.42	31.61	30.41	28.10	26.60	25.23	24.78	24.75	26.11	25.30	26.25	23.93
Molise	31.53	29.50	34.56	32.61	31.76	27.16	27.37	27.32	28.92	29.36	30.37	26.04
Campania	36.08	35.73	34.73	33.74	33.00	32.14	31.79	31.11	31.51	32.23	30.40	31.83
Puglia	32.14	31.17	29.89	30.17	28.03	27.05	26.61	26.27	26.49	26.78	25.28	25.55
Basilicata	32.74	31.65	33.35	32.82	29.16	28.42	27.08	28.04	26.41	29.24	26.52	28.76
Calabria	34.99	35.14	34.78	33.92	32.69	31.77	31.38	31.73	30.43	33.89	29.43	30.49
Sardegna	31.08	30.33	31.35	29.87	26.02	24.64	24.64	23.17	23.86	25.65	23.06	22.10
Sicilia	36.85	35.09	35.12	34.56	31.79	31.34	31.60	31.59	31.16	32.07	30.45	30.13

Comparison among Italian Regions and Autonomous Provinces.

**Figure 1:**
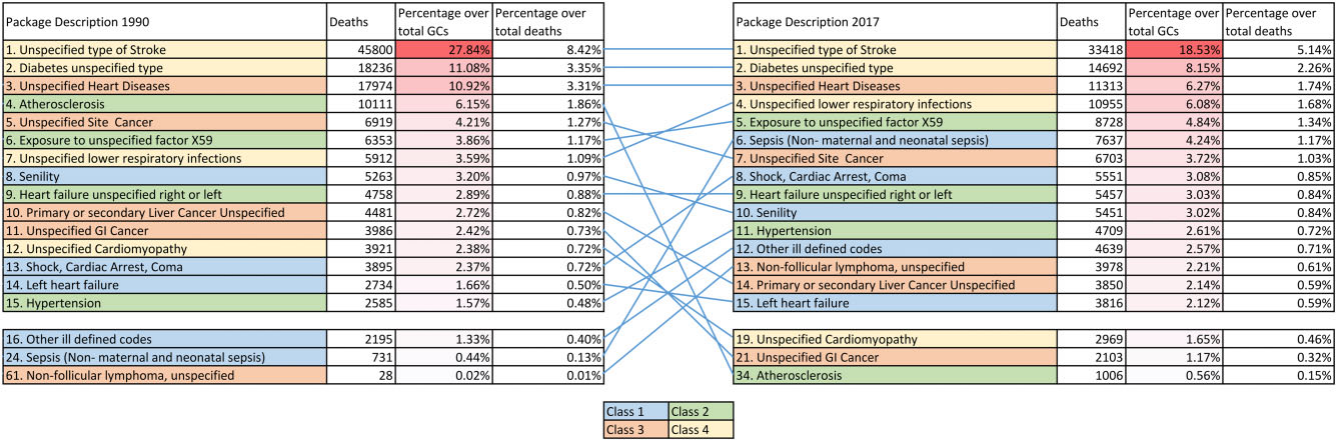
Ranking of GCs for Italy, both sexes combined, all ages, in number of deaths and percentage over the total of GCs and over total deaths per year, years 1990 and 2017

Looking at Italy and the ranking of the main GCs in 2017 compared to 1990, (figure 1) the first three GCs have remained stable since 1990, and involve two Class 4 (unspecified type of stroke and unspecified type of diabetes) and a Class 3 (unspecified heart disease). In 2017, the fourth cause was unspecified lower respiratory infections (Class 4), seventh in 1990. Exposure to unspecified factor (ICD code: X59), fifth in 2017, slightly grew since 1990 in terms of prevalence and ranking. Sepsis (excluding maternal and neonatal; Class 1) grew substantially, from 24th to 6th, and from 0.44% of all GCs identified in 1990–4.24% in 2017. Atherosclerosis (Class 2) dropped from fourth to 34th position, and from being 6.15% of all GCs in 1990–0.56% in 2017. Two other GCs saw important drops: unspecified gastrointestinal cancer (Class 3) and unspecified cardiomyopathy (Class 4). The same analysis was also performed for each of the select Western European countries (Supplementary Fig S2a–2i).

Regarding the analysis of the ranking of the main GCs in 2017 by subnational locations in Italy (Supplementary table S7), we noticed a high level of heterogeneity in the ill-defined causes and their ranking. Unspecified type of stroke remained, however, the first GC in all locations.

For Italy, the redistribution of GCs mainly affected ischemic stroke, which absorbed 27.9% of all ill-defined causes (table 3). The second UCoD affected by redistribution was Type 2 diabetes mellitus, which attracted almost 13.8% of GCs. Together with chronic ischemic heart disease (11.3%), these three UCoDs gathered more than 50% of all GCs for Italy in 2017. The same analysis was carried out for single subnational locations and is shown in Supplementary tables S8a–8u.

**Table 3:** Italy 2017, deaths added during redistribution to the 15 most affected Level 4 underlying CODs (overall deaths redistributed = 116 926)

Level 4 UCoD	Deaths before redistribution	Deaths after redistribution	Deaths added	Per cent of overall redistributed	Cumulative percentage
Ischemic stroke	10445	43049	32603	27.88	27.88
Diabetes mellitus type 2	3279	19388	16109	13.78	41.66
Chronic ischemic heart disease^a^	42180	55352	13172	11.27	52.93
Intracerebral haemorrhage	10383	18008	7625	6.52	59.45
Acute myocardial infarction^a^	24037	31041	7005	5.99	65.44
Influenza^a^	655	5755	5100	4.36	69.80
Non-Hodgkin lymphoma	948	5737	4789	4.10	73.89
Other cardiomyopathy	576	4811	4235	3.62	77.52
Pneumococcal pneumonia^a^	134	4058	3924	3.36	80.87
Other lower respiratory infections^a^	910	4303	3393	2.90	83.77
Chronic kidney disease due to hypertension	5811	8907	3096	2.65	86.42
Chronic kidney disease due to diabetes mellitus type 2	573	2720	2147	1.84	88.26
Subarachnoid haemorrhage	1770	3027	1258	1.08	89.33
Non-rheumatic calcific aortic valve disease	4083	5320	1237	1.06	90.39
Pedestrian road injuries	727	1531	805	0.69	91.08

a These causes are a further subdivision of Level 4 causes used for redistribution. In particular: ‘chronic ischemic heart disease’ and ‘acute myocardial infarction’ merge into Level 4 ‘ischemic heart disease’; ‘influenza’, ‘pneumococcal pneumonia’ and ‘other lower respiratory infections’ merge into Level 4 ‘lower respiratory infections’.

Supplementary table S9 shows how the 15 main GCs (by number of attributed deaths) were redistributed to UCoDs (the year 2017). If, for each GC, the UCoDs were 10 or less, these were all reported. Otherwise, we only reported the first 10 by number of redistributed deaths. The list of UCoDs is short, as in the case of Class 4 GCs like ‘diabetes unspecified type’, or long, namely for Class 1 GCs, such as ‘sepsis’ or ‘shock, cardiac attack, coma’.

The 15 UCoDs affected by redistribution can give an idea of how ill-defined causes can affect reporting (Supplementary table S10). There is some obvious specularity with Supplementary table S9. Ischemic stroke is the primary cause most affected by redistribution, with 84% of deaths redistributed to this cause coming from ‘unspecified type of stroke’. Diabetes mellitus Type 2, which is the second most affected underlying cause, receives 91% of its redistributions from ‘unspecified type of diabetes’. The third cause, ischemic heart disease, receives 35% from ‘unspecified heart disease’, but also 9% from ‘unspecified left or right heart failure’ and ‘unspecified cardiovascular disease’. Intracerebral haemorrhage, the fourth primary CoD affected, receives 66% of the redistributed deaths from ‘unspecified type of stroke’, and 12% from ‘hypertension’.

## Discussion

The present analysis provides new insights on why official VR data can be different from GBD estimates. The comparison between Italy and other Western European countries, and among Italian subnational locations, reveals strengths and weaknesses in current reporting systems. Despite realities with lower percentages of overall GCs can set an achievable standard, less performing countries and locations still show lower percentages for specific GCs and should be carefully studied.

GCs affect, albeit to a different extent, all countries and VR systems. All countries considered in the present analysis—with the exception of Sweden which had the lowest level of GCs in 1990—saw a reduction in the percentage of GCs over total deaths. However, none of the countries achieved percentages lower than 25% for all ages and 22% for age-standardized rates.

The range in the overall percentage of GCs encountered in the comparison by country is almost the same as that found comparing Italian subnational locations. Throughout the 10 countries considered, in 2016/2017, the age-standardized proportion of GCs varied between 22% for the UK to 33% for Greece. In Italy, it varied between 20% for Bolzano and 32% for Campania. This implies the margins of improvement are potentially wide. Despite the possible differences in national reporting systems, it is important to note that all Italian regions follow the same system and differences cannot be attributed to this aspect.

Wide heterogeneity exists in the ranking of the single GCs by comparing different countries or subnational Italian locations. As mentioned, even less performing countries and subnational locations have good performances on certain GCs, meaning that we could all learn from others. Greece has the lowest proportion of ‘unspecified heart disease’ and ‘unspecified cardiovascular disease’ across the considered countries (Supplementary table S4), while Portugal has the lowest proportion of ‘undetermined intent poisoning by multiple or unspecified drug’, which is the ninth GC in the UK. The same occurs in Italy, with Sicily being the location with the lowest proportion of ‘sepsis’, and Calabria having the lowest proportion of ‘unspecified non-follicular lymphoma’ (Supplementary table S7).

The inconsistency in the percentage composition of Classes 1 and 2 vs. Classes 3 and 4, with respect to the overall proportion of GCs, across countries and locations, suggests that, despite being relatively easier to reduce, Classes 3 and 4 GCs have not been systematically tackled even in better performing countries and locations. The lack of a significant decrease in Classes 1 and 2 GCs, highlights a serious difficulty in dealing with this type of GCs, but also a lack of systematic action in finding solutions for the reduction of these which are the most difficult GCs to redistribute.

Heterogeneity in GCs across countries and locations could be explained, at least partially, as a result of different epidemiological profiles. However, by comparing the age-standardized death rate for stroke for Italy (34.9 per 100k) and Spain (29.1)—according to GBD 2019—and the proportion of the GC ‘unspecified type of stroke’ from Supplementary table S4, being 5.1% for Italy and 2.5% for Spain, we are led to believe there is significant room for improvement for Italy. This being corroborated by the fact that the Province of Trento, with 2.7%, has almost reached the level of Spain already (Supplementary table S7).

Regarding ‘unspecified type of diabetes’, the second GC for Italy (2.3%), we could find no objective difficulty in the determination of the actual type of diabetes as a CoD.

Despite the measures adopted to improve coding following international standard rules, a share of GCs remains, as a consequence of errors in medical certifications rather than in the coding process. The quality of mortality statistics in Italy is deemed very high and, according to the WHO, which uses different definitions and grouping processes with respect to GBD, the proportion of GCs was the lowest among high-income countries.^17^ However, this proportion is not negligible. With Italy representing one of the top-rated Health Systems, we find it hard to justify having every year more than 30 000 deaths attributed to unspecified stroke (Class 4), 15 000 to unspecified type of diabetes (Class 4), 10 000 to unspecified heart disease (Class 3), 8000 to X59-exposure to unspecified factor causing fracture or other unspecified injury (Class 2), and 7000 to sepsis (Class 1).

Death certification is a complex task, but systems can be improved. Local training of medical doctors and constant review of records at hospital and local level, focusing on most common GCs and less performing regions, would be needed. Electronic CoD registration systems can help physicians reduce errors and increase precision in data entry,^4^^,^^18^ while increasingly accurate algorithms of redistribution could improve GBD estimates.

In the current pandemic context, with the urge to accelerate the process of acquisition of the CoDs, the Italian Ministries of Economy and Finance, Health, and Internal Affairs drafted a Decree introducing digital reporting performed directly by the certifying doctors.^19^ The decree is now under the scrutiny of the Data Protection Authority, the Regions and the National Association of Italian Municipalities (ANCI).

Although the GBD identifies GCs and defines redistributions by sex and age, the main limitation of our analysis is the lack of consideration of sex and age differences. Finally, a detailed analysis of national CoD registration systems could have also been of interest. Our concern, however, was that the inclusion of these dimensions would have caused too much dispersion. The different timing in the implementation of ICD-10 updates among countries has likely affected the comparability among countries and, within a given country, throughout time. In this regard, in Italy, the adoption of the 2016 version of the ICD-10 and of a new automated coding system (Iris software) caused some time trend comparability issues.^20^

This thorough analysis of GCs conducted for Italy, with a comparision with select Western European countries, should be considered as a first step towards structured actions for the improvement of CoD classifications in Italy, with an alignment of all regions toward the best achievable standards.

## Supplementary data

Supplementary data are available at *EURPUB* online.

## Acknowledgement

**GBD 2019 Italy Garbage Codes Collaborators**

Lorenzo Monasta,^1^ Gianfranco Alicandro,^2,3^ Maja Pasovic,^4^ Matthew Cunningham,^4^ Benedetta Armocida,^1^ Luciana Albano,^5^ Ettore Beghi,^6^ Massimiliano Beghi,^7^ Cristina Bosetti,^8^ Nicola Luigi Bragazzi,^9^ Giulia Carreras,^10^ Giulio Castelpietra,^11,12^ Prof Alberico L. Catapano,^13,14^ Maria Sofia Cattaruzza,^15^ Giulia Collatuzzo,^16^ Sara Conti,^17^ Giovanni Damiani,^18,19^ Pietro Ferrara,^20^ Carla Fornari,^17^ Silvano Gallus,^21^ Simona Giampaoli,^22^ Davide Golinelli,^23^ Prof Gaetano Isola,^24^ Paolo Lauriola,^25^ Prof Carlo La Vecchia,^26^ Matilde Leonardi,^27^ Francesca Giulia Magnani,^27^ Giada Minelli,^28^ Marcello Moccia,^29^ Paolo Pedersini,^30^ Norberto Perico,^31^ Alberto Raggi,^27^ Prof Giuseppe Remuzzi,^31^ Francesco Sanmarchi,^23^ Davide Sattin,^32^ Brigid Unim,^33^ Jorge Hugo Villafañe,^34^ Prof Francesco S Violante,^16,35^ Prof Christopher J L Murray,^4,36^ Luca Ronfani,^1^ Prof Mohsen Naghavi.^4,36^

^1^Institute for Maternal and Child Health IRCCS “Burlo Garofolo”, Trieste, Italy; ^2^Department of Pathophysiology and Transplantation, University of Milan Bicocca (Università degli Studi di Milano), Milan, Italy; ^3^Cystic Fibrosis Center, Fondazione IRCCS Ospedale Maggiore Policlinico (IRCCS "Ca' Granda Maggiore Policlinico" Hospital Foundation), Milan, Italy; ^4^Institute for Health Metrics and Evaluation, University of Washington, Seattle, WA, USA; ^5^Department of Experimental Medicine, University of Campania Luigi Vanvitelli, Naples, Italy; ^6^Department of Neuroscience, Mario Negri Institute for Pharmacological Research, Milan, Italy; ^7^Department of Mental Health, AUSL Romagna, Ravenna, Italy; ^8^Department of Oncology, Mario Negri Institute for Pharmacological Research, Milan, Italy; ^9^University of Genoa, Genoa, Italy; ^10^Institute for Cancer Research, Prevention and Clinical Network, Florence, Italy; ^11^Department of Medicine, University of Udine, Udine, Italy; ^12^Department of Mental Health, Healthcare Agency "Friuli Occidentale", Pordenone, Italy; ^13^Department of Pharmacological and Biomolecular Sciences, University of Milan, Milan, Italy; ^14^MultiMedica, IRCCS, Milano, Italy; ^15^Department of Public Health and Infectious Diseases, La Sapienza University, Rome, Italy; ^16^Department of Medical and Surgical Sciences, University of Bologna, Bologna, Italy; ^17^School of Medicine and Surgery, University of Milan Bicocca, Monza, Italy; ^18^IRCCS Istituto Ortopedico Galeazzi (Galeazzi Orthopedic Institute IRCCS), University of Milan, Milan, Italy; ^19^Department of Dermatology, Case Western Reserve University, Cleveland, OH, USA; ^20^Research Center on Public Health, University of Milan Bicocca, Monza, Italy; ^21^Department of Environmental Health Sciences, Mario Negri Institute for Pharmacological Research, Milan, Italy; ^22^Department of Cardiovascular Endocrine-metabolic Diseases and Aging, Istituto Superiore di Sanità, Rome, Italy; ^23^Department of Biomedical and Neuromotor Sciences, University of Bologna, Bologna, Italy; ^24^Department of General Surgery and Surgical-Medical Specialties, University of Catania, Catania, Italy; ^25^International Society Doctors for the Environment, Arezzo, Italy; ^26^Department of Clinical Sciences and Community Health, University of Milan, Milan, Italy; ^27^UO Neurologia, Salute Pubblica e Disabilità, Fondazione IRCCS Istituto Neurologico Carlo Besta (Neurology, Public Health and Disability Unit, Carlo Besta Neurological Institute), Milan, Italy; ^28^Unit of Statistics, Istituto Superiore di Sanità, Rome, Italy; ^29^Department of Neurosciences, Federico II University, Naples, Italy; ^30^Clinical Research Department, IRCCS Fondazione Don Carlo Gnocchi, Milan, Italy; ^31^Mario Negri Institute for Pharmacological Research, Bergamo, Italy; ^32^IRCCS Istituti Clinici Scientifici Maugeri (IRCCS Maugeri Scientific Clinical Institute), Milan, Italy; ^33^Department of Cardiovascular, Endocrine-metabolic Diseases and Aging, National Institute of Health, Rome, Italy; ^34^Clinical Research Department, IRCCS Fondazione Don Carlo Gnocchi, Milan, Italy; ^35^Occupational Health Unit, Sant'Orsola Malpighi Hospital, Bologna, Italy; ^36^Department of Health Metrics Sciences, School of Medicine, University of Washington, Seattle, WA, USA.

## Funding

The GBD 2019 study was supported by the Bill & Melinda Gates Foundation. The funder of the study had no role in study design, data collection, data analysis, data interpretation, writing of the present report or decision to publish. The corresponding author had full access to all the data in the study and had final responsibility for the decision to submit for publication.

*Conflicts of interest*: A L Catapano reports grants or contracts from Sonofi, El Lilly, Mylan, Sanofi Regeneron, Menarini, and Amgen; payment or honoraria for lectures, presentations, speakers bureaus, manuscript writing or educational events from Amgen, AstraZeneca, Aegerion, Amryt, Daiichi Sankya, Esperion, Kowa, Ionis Pharmaceuticals, Mylan, Merck, Menarini, Novartis, Recordati, Regeneron, Sandoz, and Sanofi; all outside the submitted work. D Golinelli reports a grant from the Italian Ministry of Education as payments to their institution; consulting fees from ECM SRL for a consultation for Medical Device CE marking; all outside the submitted work. M Moccia reports grants from MAGNIMS-ECTRIMS, UK-MS Society, and Merck; consulting fees from Ipsen, Sanofi-Genzyme, and Merck; honoraria for a speaker activity from Merck, Roche, and Sanofi-Genzyme; travel support for attending meetings from Merck, Biogen, and Sanofi-Genzyme; participation on an Advisory Board with Merck and Roche; all outside the submitted work. M Pasovic reports support for the present manuscript from the Bill and Melinda Gates foundation through their employment at IHME. N Perico reports participation on a Data Safety Monitoring Board or Advisory Board with Bayer AG, outside the submitted work. G Remuzzi reports consulting fees from Akebia Pharmaceuticals, Alexion Pharmaceuticals, AstraZeneca, Menarini Ricerche Spa, Janssen R&D, and BioCryst; travel support from Boehringer; all outside the submitted work

Key points

- The proportion of reported ill-defined and trivial causes of death can reach over 30%, even in high-income countries and in countries with high-performing health systems, seriously affecting evidence-based health planning.
- The Global Burden of Disease Study identifies these ‘garbage codes’, and redistributes them to underlying causes of death using evidence-based algorithms.
- The redistribution generates discrepancies between deaths allocated to specific underlying causes of death if we compare vital registration system data and GBD estimates.
- Our comparative analysis of garbage codes and on the effects of redistribution, among subnational locations for Italy, and select Western European countries, shows a high degree of heterogeneity that can be used to identify areas of potential improvement.

## Supplementary Material

ckab194_Supplementary_Data
