# Corrigendum to: Redistribution of garbage codes to underlying causes of death: a systematic analysis on Italy and a comparison with most populous Western European countries based on the Global Burden of Disease Study 2019

**DOI:** 10.1093/eurpub/ckac021

**Published:** 2022-03-12

*European Journal of Public Health*, 2021; https://doi.org/10.1093/eurpub/ckab194

In the originally published version of this manuscript, an author was erroneously omitted from the list of authors. The list should read: “Lorenzo Monasta, Gianfranco Alicandro, Maja Pasovic, Matthew Cunningham, Benedetta Armocida, Christopher J L Murray, Luca Ronfani, Mohsen Naghavi, GBD 2019 Italy Causes of Death Collaborators” instead of “Lorenzo Monasta, Gianfranco Alicandro, Maja Pasovic, Matthew Cunningham, Benedetta Armocida, Luca Ronfani, Mohsen Naghavi, GBD 2019 Italy Causes of Death Collaborators”. This error has been corrected online.

