# Supplementary material for: Redistribution of garbage codes to underlying causes of death: a systematic analysis on Italy and a comparison with most populous Western European countries based on the Global Burden of Disease Study 2019
Source: Eur J Public Health. 2022 Jan 21;32(3):456–62. doi: 10.1093/eurpub/ckab194 (PMC9159332; doi:10.1093/eurpub/ckab194)
Supplement: ckab194_Supplementary_Data [file ckab194_supplementary_data.zip › Italy Garbage-Supplement-211207.pdf]

Supplementary Annex to "Redistribution of garbage codes to underlying causes of death: a systematic analysis on Italy and a comparison with most populous Western European countries based on the Global Burden of Disease Study 2019."

Supplementary Table 1. Percentage of "garbage codes" over all causes of death reported, all ages, both sexes. Comparison among most Western European countries with more than 10 million inhabitants.

	1990	1995	2000	2005	2010	2011	2012	2013	2014	2015	2016	2017
Germany	32.38%	30.16%	29.44%	27.28%	26.64%	25.51%	25.77%	25.89%	25.44%	26.19%	25.62%	25.68%
**Italy**	**31.70%**	**30.86%**	**30.47%**	**29.52%**	**28.27%**	**27.79%**	**28.16%**	**28.10%**	**28.13%**	**29.50%**	**27.11%**	**27.71%**
Sweden	26.18%	26.45%	27.88%	27.37%	29.64%	29.61%	30.11%	29.28%	28.73%	29.92%	29.56%	29.19%
United Kingdom	26.47%	28.94%	30.83%	28.64%	27.15%	27.37%	28.11%	28.47%	27.88%	29.44%	29.12%	29.98%
Spain	39.50%	35.75%	34.99%	34.89%	31.04%	31.33%	32.00%	30.37%	30.68%	32.54%	30.17%	30.82%
Netherlands	31.69%	33.51%	36.21%	34.62%	32.79%	31.95%	32.89%	34.27%	33.76%	33.99%	34.34%	34.51%
Belgium	35.46%	35.63%	34.10%	33.87%	34.88%	35.08%	36.35%	35.96%	35.71%	36.33%	36.14%	
France	38.96%	38.88%	38.04%	36.69%	37.05%	36.79%	38.14%	38.19%	36.53%	37.78%	37.89%	
Portugal	54.72%	54.09%	53.49%	51.47%	44.51%	43.48%	44.06%	42.41%	39.01%	39.57%	39.08%	38.60%
Greece	52.84%	51.46%	50.91%	48.58%	44.85%	44.78%	45.08%	41.72%	41.03%	41.28%	40.48%	

Supplementary Table 2. For Class 1 and 2 garbage code packages, percentage of garbage codes over all causes of death reported, age-standardized.

	1990		1995		2000		2005		2010	2011	2012	2013	2014	2015	2016	2017
United Kingdom	6.49%		7.85%		8.62%		8.50%		8.69%	8.42%	8.33%	8.55%	8.29%	8.77%	8.72%	8.81%
**Italy**	**10.62%**		**10.81%**		**11.13%**		**10.22%**		**10.17%**	**10.28%**	**10.88%**	**11.00%**	**11.13%**	**11.84%**	**10.99%**	**11.65%**
Spain	17.88%		14.22%		14.21%		14.16%		13.13%	13.49%	13.22%	12.16%	12.07%	12.79%	12.27%	12.44%
Sweden	9.85%		10.70%		11.35%		11.40%		15.78%	15.04%	14.03%	13.53%	13.24%	14.69%	13.92%	13.36%
Portugal	23.22%		22.31%		24.57%		24.33%		21.01%	19.38%	20.42%	18.97%	14.05%	14.56%	14.47%	14.36%
Netherlands	13.69%		14.80%		15.84%		14.86%		14.17%	14.02%	13.80%	15.00%	14.61%	14.29%	14.51%	14.36%
Germany	16.94%		13.92%		14.94%		14.63%		14.91%	14.23%	14.42%	14.57%	14.63%	15.23%	15.15%	14.96%
Greece	23.81%		20.90%		21.76%		20.42%		16.38%	16.38%	16.37%	15.53%	17.61%	17.70%	16.47%	
Belgium	19.45%		15.61%		13.94%		14.05%		17.37%	16.89%	18.21%	17.46%	17.40%	17.73%	17.65%	
France	19.38%		18.43%		17.69%		17.64%		19.32%	19.13%	19.96%	20.61%	19.49%	19.82%	19.98%	

Supplementary Table 3. For Class 3 and 4 garbage code packages, percentage of garbage codes over all causes of death reported, age-standardized.

	1990		1995		2000		2005		2010	2011	2012	2013	2014	2015	2016	2017
Germany	13.26%		14.92%		13.99%		12.58%		11.12%	10.49%	10.28%	10.22%	9.87%	9.69%	9.60%	9.82%
Sweden	13.06%		12.43%		11.74%		11.07%		9.76%	10.40%	10.85%	10.33%	10.07%	10.16%	9.64%	9.56%
Netherlands	16.47%		15.59%		15.32%		14.36%		12.83%	12.36%	12.37%	12.01%	11.70%	12.08%	11.76%	11.54%
Spain	20.65%		19.39%		16.19%		15.56%		13.13%	13.10%	13.66%	13.26%	13.21%	12.91%	11.78%	11.87%
France	17.26%		17.23%		16.22%		14.76%		12.69%	12.40%	12.16%	11.84%	11.80%	12.04%	12.08%	
United Kingdom	18.81%		18.47%		18.67%		17.38%		15.77%	14.64%	14.27%	14.10%	13.41%	13.52%	13.08%	13.26%
Belgium	12.57%		16.72%		16.22%		15.59%		13.80%	13.92%	13.38%	13.36%	13.26%	13.31%	13.14%	
**Italy**	**20.61%**		**20.09%**		**19.10%**		**19.12%**		**17.44%**	**16.10%**	**15.73%**	**15.52%**	**15.47%**	**15.58%**	**14.59%**	**14.16%**
Greece	23.17%		24.87%		23.25%		22.25%		20.88%	20.42%	19.85%	18.41%	17.25%	16.21%	16.68%	
Portugal	28.13%		28.14%		25.24%		24.52%		20.80%	20.82%	20.39%	18.88%	18.44%	18.09%	18.31%	16.70%

Supplementary Figure 1. Repartition between Class 1 and 2, and Class 3 and 4 of garbage codes over all causes of death reported, age-standardized, both sexes, most recent available year between 2016 and 2017. Most populated Western European countries.

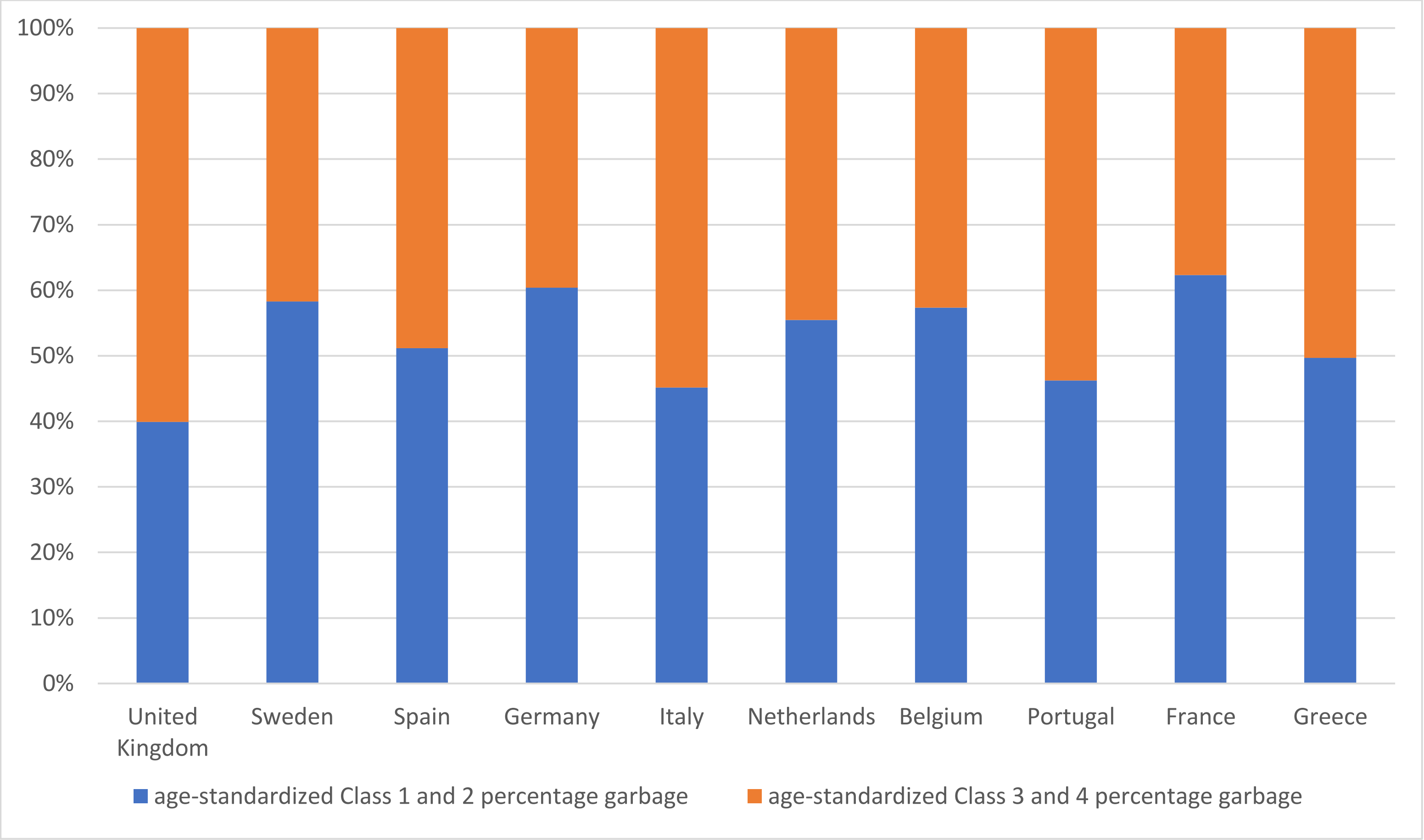

Supplementary Table 4. Ten main Garbage Codes packages, considered as percentage over the total deaths for 2017 when available (or 2016 otherwise), ordered by ranking for Italy.

**Package Description***	**Italy**	**Belgium**	**France**	**Germany**	**Greece**	**Netherlands**	**Portugal**	**Spain**	**Sweden**	**United Kingdom**
**Unspecified type of Stroke**	5.14% (1)	3.44% (3)	2.59% (4)	2.58% (3)	8.42% (1)	3.52% (2)	7.93% (1)	2.53% (4)	3.20% (2)	3.61% (2)
**Diabetes unspecified type**	2.26% (2)	0.84% (14)	1.20% (8)	1.35% (5)	0.88% (10)	1.18% (7)	1.57% (6)	1.54% (6)	1.31% (5)	0.38% (14)
**Unspecified Heart Diseases**	1.74% (3)	1.00% (9)	0.60% (14)	0.30% (22)	0.17% (23)	0.26% (25)	0.19% (31)	0.37% (21)	0.35% (19)	0.21% (22)
**Unspecified lower respiratory infections**	1.68% (4)	3.86% (1)	2.36% (5)	1.97% (4)	4.66% (3)	2.54% (4)	4.86% (2)	2.58% (3)	2.02% (4)	5.95% (1)
**Exposure to unspecified factor X59**	1.34% (5)	0.91% (10)	1.72% (6)	0.38% (17)	0.33% (19)	0.39% (19)	0.66% (13)	0.41% (19)	1.00% (7)	0.45% (12)
**Sepsis (Non- maternal and neonatal sepsis)**	1.17% (6)	0.84% (13)	0.91% (10)	0.83% (7)	3.05% (5)	1.07% (9)	0.92% (11)	0.94% (9)	0.90% (9)	0.52% (10)
**Unspecified Cancer Site**	1.03% (7)	1.26% (7)	1.53% (7)	1.28% (6)	1.48% (7)	1.51% (5)	1.07% (9)	1.07% (7)	1.23% (6)	1.56% (3)
**Shock, Cardiac Arrest, Coma**	0.85% (8)	2.22% (5)	3.72% (2)	0.36% (18)	4.55% (4)	1.41% (6)	1.49% (7)	0.31% (24)	0.36% (17)	0.02% (59)
**Heart failure unspecified right or left**	0.84% (9)	3.85% (2)	2.87% (3)	3.43% (1)	5.82% (2)	4.42% (1)	3.62% (3)	4.00% (1)	3.52% (1)	0.22% (21)
**Senility**	0.84% (10)	1.40% (6)	0.67% (13)	0.19% (26)	1.12% (8)	1.15% (8)	1.20% (8)	0.43% (17)	0.94% (8)	1.45% (4)
Hypertension	0.72% (11)	0.32% (24)	0.73% (11)	0.77% (9)	0.81% (11)	0.43% (17)	0.70% (12)	0.85% (10)	0.83% (10)	0.27% (17)
Other ill-defined causes of death	0.71% (12)	3.32% (4)	5.88% (1)	2.78% (2)	0.09% (33)	3.30% (3)	2.66% (4)	2.20% (5)	2.21% (3)	1.26% (5)
Left heart failure	0.59% (15)	1.23% (8)	0.73% (12)	0.77% (10)	1.67% (6)	0.83% (10)	1.03% (10)	1.02% (8)	0.20% (26)	0.70% (6)
Acute kidney failure	0.58% (16)	0.85% (12)	0.53% (18)	0.79% (8)	0.71% (13)	0.52% (14)	0.43% (18)	0.70% (11)	0.23% (24)	0.13% (25)
Unspecified cardiovascular diseases	0.57% (17)	0.33% (23)	0.16% (36)	0.02% (65)	0.00% (85)	0.29% (23)	0.18% (32)	0.10% (37)	0.42% (15)	0.05% (43)
Cardiac rhythm disorders	0.52% (18)	0.85% (11)	1.14% (9)	0.54% (13)	0.14% (27)	0.52% (15)	0.28% (27)	0.54% (13)	0.32% (21)	0.08% (30)
Unspecified gastrointestinal cancer	0.32% (21)	0.52% (17)	0.43% (21)	0.18% (27)	0.38% (17)	0.80% (11)	0.29% (26)	0.23% (27)	0.30% (22)	0.60% (7)
Unspecified chronic respiratory diseases	0.26% (23)	0.21% (31)	0.27% (32)	0.06% (43)	0.01% (62)	0.36% (21)	1.62% (5)	3.27% (2)	0.11% (34)	0.09% (28)
Pneumonitis	0.20% (27)	0.69% (15)	0.57% (14)	0.34% (20)	1.01% (9)	0.18% (29)	0.54% (15)	0.51% (14)	0.12% (31)	0.36% (16)
Undetermined intent Poisoning by multiple or unspecified drug	0.08% (42)	0.18% (33)	0.30% (28)	0.16% (29)	0.18% (22)	0.16% (31)	0.06% (47)	0.20% (29)	0.55% (13)	0.56% (9)
Alcoholic hepatic failure	0.03% (57)	0.06% (52)	0.04% (64)	0.07% (41)	0.02% (57)	0.07% (43)	0.22% (29)	0.08% (42)	0.10% (35)	0.58% (8)

* In bold, the first ten for Italy. The other packages have been included to consider the first ten packages for all considered countries. On the right of each percentage column, the ranking for the country.

Supplementary Table 5. Percentage of Class 1 and 2 garbage codes over all causes of death reported, age-standardized. Comparison among Italian Regions and Autonomous Provinces

**Sub-national location name**	1990	1995	2000	2005	2010	2011	2012	2013	2014	2015	2016	2017
Piemonte	10.70%	10.34%	10.46%	8.95%	9.15%	9.12%	9.76%	9.95%	9.91%	10.43%	9.41%	9.79%
Valle d'Aosta	14.05%	11.52%	9.45%	7.19%	11.33%	8.07%	7.43%	10.50%	8.33%	9.76%	7.30%	8.91%
Lombardia	9.81%	8.87%	9.45%	8.53%	8.79%	8.79%	8.97%	9.73%	9.56%	10.17%	9.42%	9.85%
Provincia autonoma di Bolzano	11.30%	10.01%	12.24%	8.38%	8.47%	7.13%	9.89%	9.70%	11.21%	10.14%	8.43%	9.93%
Provincia autonoma di Trento	9.38%	11.52%	8.65%	9.58%	7.28%	8.59%	8.71%	10.55%	12.35%	12.22%	11.49%	10.15%
Veneto	9.92%	10.31%	10.83%	10.49%	9.14%	9.45%	9.92%	10.90%	10.51%	10.64%	10.19%	10.62%
Friuli-Venezia Giulia	12.21%	9.86%	9.59%	9.46%	9.23%	9.38%	9.94%	9.45%	10.64%	10.38%	9.99%	9.16%
Liguria	11.37%	13.67%	15.12%	14.38%	12.82%	12.58%	12.47%	11.80%	12.71%	13.61%	14.25%	14.61%
Emilia-Romagna	9.65%	9.64%	9.67%	8.82%	8.76%	8.68%	9.41%	9.85%	9.79%	10.37%	10.04%	10.76%
Toscana	11.54%	11.12%	10.52%	9.62%	10.03%	10.22%	11.65%	10.93%	10.93%	11.71%	10.41%	11.61%
Umbria	9.62%	9.21%	10.00%	9.02%	9.08%	10.10%	9.88%	11.61%	10.16%	11.46%	11.54%	11.24%
Marche	9.65%	9.59%	10.49%	9.55%	8.86%	9.18%	9.78%	8.77%	9.81%	10.73%	8.84%	9.64%
Lazio	9.20%	10.48%	11.49%	10.21%	10.76%	10.55%	11.18%	10.84%	11.44%	12.26%	10.52%	11.52%
Abruzzo	11.69%	12.62%	12.57%	10.25%	10.00%	9.77%	9.92%	10.75%	10.84%	10.62%	11.72%	10.19%
Molise	10.02%	11.24%	13.50%	13.69%	12.20%	10.73%	11.88%	11.18%	13.30%	13.35%	15.71%	12.48%
Campania	11.51%	12.85%	13.08%	12.46%	12.95%	13.33%	13.80%	13.19%	13.40%	14.44%	13.95%	15.34%
Puglia	9.73%	10.53%	10.07%	9.56%	9.61%	9.89%	10.54%	10.63%	10.62%	11.70%	10.48%	10.83%
Basilicata	10.86%	11.34%	12.18%	9.86%	9.51%	10.17%	10.43%	11.30%	9.94%	11.44%	11.98%	13.66%
Calabria	12.33%	13.74%	14.24%	13.17%	12.41%	12.81%	13.02%	13.31%	13.38%	14.96%	12.92%	14.50%
Sardegna	11.09%	10.40%	10.23%	10.41%	8.97%	9.12%	9.68%	9.68%	10.28%	11.56%	9.92%	10.01%
Sicilia	12.74%	12.38%	12.69%	11.86%	11.36%	12.00%	12.78%	12.85%	12.95%	13.93%	13.17%	13.76%

Supplementary Table 6. Percentage of Class 3 and 4 “garbage codes” over all causes of death reported, age-standardized. Comparison among Italian Regions and Autonomous Provinces

**Sub-national location name**	1990	1995	2000	2005	2010	2011	2012	2013	2014	2015	2016	2017
Piemonte	20.35%	20.28%	18.82%	18.98%	17.51%	15.95%	15.78%	15.66%	15.49%	15.97%	13.83%	14.61%
Valle d'Aosta	16.80%	17.34%	16.90%	16.08%	15.91%	13.38%	11.49%	15.91%	12.83%	16.05%	11.88%	12.93%
Lombardia	19.54%	19.77%	17.91%	18.29%	16.10%	15.50%	14.94%	14.50%	14.65%	15.03%	14.45%	13.52%
Provincia autonoma di Bolzano	17.55%	16.40%	14.05%	12.51%	11.80%	10.40%	10.67%	12.51%	10.71%	10.53%	10.50%	10.56%
Provincia autonoma di Trento	16.97%	16.40%	14.46%	15.93%	11.85%	10.08%	11.97%	14.60%	13.91%	12.95%	11.36%	13.42%
Veneto	17.19%	16.77%	17.32%	17.20%	15.96%	14.36%	14.88%	14.28%	13.85%	14.11%	13.34%	13.06%
Friuli-Venezia Giulia	17.46%	18.56%	16.37%	17.44%	15.63%	14.66%	14.65%	14.51%	13.51%	15.80%	13.35%	13.90%
Liguria	20.23%	21.00%	18.05%	18.54%	16.44%	14.34%	15.31%	14.41%	14.99%	14.43%	13.84%	13.12%
Emilia-Romagna	18.80%	18.65%	18.25%	17.55%	16.01%	13.75%	14.30%	14.18%	14.60%	13.99%	13.42%	12.62%
Toscana	21.05%	20.57%	19.79%	19.61%	17.94%	15.87%	15.78%	15.54%	15.51%	14.86%	15.34%	13.35%
Umbria	20.49%	19.04%	17.95%	18.04%	15.77%	13.90%	14.30%	14.87%	14.77%	14.78%	14.50%	12.30%
Marche	19.92%	18.81%	18.06%	18.92%	18.60%	15.44%	14.82%	14.36%	14.34%	14.64%	13.13%	12.48%
Lazio	18.79%	19.37%	18.36%	18.27%	16.64%	15.51%	14.52%	14.71%	14.90%	15.21%	13.92%	13.88%
Abruzzo	20.72%	18.99%	17.84%	17.85%	16.59%	15.46%	14.86%	14.00%	15.28%	14.68%	14.53%	13.74%
Molise	21.51%	18.26%	21.06%	18.92%	19.56%	16.43%	15.49%	16.14%	15.62%	16.01%	14.66%	13.57%
Campania	24.58%	22.88%	21.65%	21.28%	20.06%	18.82%	17.99%	17.92%	18.11%	17.79%	16.45%	16.48%
Puglia	22.40%	20.64%	19.83%	20.61%	18.42%	17.16%	16.06%	15.64%	15.87%	15.08%	14.80%	14.72%
Basilicata	21.88%	20.31%	21.16%	22.97%	19.65%	18.25%	16.65%	16.74%	16.46%	17.80%	14.55%	15.10%
Calabria	22.65%	21.40%	20.53%	20.75%	20.28%	18.96%	18.37%	18.43%	17.05%	18.92%	16.51%	15.99%
Sardegna	19.99%	19.92%	21.12%	19.46%	17.04%	15.52%	14.96%	13.49%	13.58%	14.09%	13.15%	12.09%
Sicilia	24.11%	22.71%	22.43%	22.70%	20.43%	19.34%	18.81%	18.74%	18.22%	18.14%	17.27%	16.37%

Supplementary Figure 2a. Ranking of Garbage Codes for Belgium, both sexes combined, all ages, in number of deaths and percentage over the total of Garbage Codes and over total deaths per year, years 1990 and 2016.

Package Description 1990	Deaths	Percentage over total GCs	Percentage over total deaths
1. Unspecified type of Stroke	6702	18.15%	6.44%
2. Left Heart Failure	3067	8.31%	2.95%
3. Flutter and Fibrillation	2409	6.52%	2.31%
4. Unspecified sign & symptom for Heart diseases	2274	6.16%	2.18%
5. Unspecified site Cancer	2094	5.67%	2.01%
6. Atherosclerosis	1932	5.23%	1.86%
7. Cardiac Diseases other	1844	4.99%	1.77%
8. Diabetes unspecified type	1714	4.64%	1.65%
9. Senility	1529	4.14%	1.47%
10. Other Ill Defined code for causes of death	1027	2.78%	0.99%
11. Shock, Cardiac Arrest, Coma	1010	2.73%	0.97%
12. Respiratory failure	910	2.46%	0.87%
13. CKD	895	2.42%	0.86%
14. Arritmia	885	2.40%	0.85%
15. Cardiac Diseases and Pulmonary embolism	815	2.21%	0.78%

Package Description 2016	Deaths	Percentage over total GCs	Percentage over total deaths
1. Unspecified lower respiratory infection	4171	10.68%	3.86%
2. Heart failure unspecified right or left	4159	10.64%	3.85%
3. Unspecified type of Stroke	3713	9.50%	3.44%
4. Other, Ill Defined code for causes of death	3584	9.17%	3.32%
5. Shock, Cardiac Arrest, Coma	2403	6.15%	2.22%
6. Senility	1514	3.87%	1.40%
7. Unspecified Site Cancer	1360	3.48%	1.26%
8. Left Heart Failure	1331	3.41%	1.23%
9. Unspecified Heart Diseases	1086	2.78%	1.00%
10. Exposure to unspecified factor X59	985	2.52%	0.91%
11. Cardiac rhythm disorders	923	2.36%	0.85%
12. Acute kidney failure	919	2.35%	0.85%
13. Sepsis (Non- maternal and neonatal sepsis)	907	2.32%	0.84%
14. Diabetes unspecified type	905	2.32%	0.84%
15. Pneumonitis	751	1.92%	0.69%

Class 1	Class 2
Class 3	Class 4

Supplementary Figure 2b. Ranking of Garbage Codes for France, both sexes combined, all ages, in number of deaths and percentage over the total of Garbage Codes and over total deaths per year, years 1990 and 2016.

Package Description 1990	Deaths	Percentage over total GCs	Percentage over total deaths
1. Unspecified type of Stroke	30669	14.96%	5.83%
2. Heart failure unspecified right or left	17407	8.49%	3.31%
3. Shock, Cardiac Arrest, Coma	14083	6.87%	2.68%
4. Other Ill Defined code for causes of death	12066	5.89%	2.29%
5. Exposure to unspecified factor X59	11377	5.55%	2.16%
6. Unspecified lower respiratory infection	11210	5.47%	2.13%
7. Unspecified Site Cancer	9844	4.80%	1.87%
8. Left heart failure	9527	4.65%	1.81%
9. Motor vehicle accident of unspecified nature	8927	4.35%	1.70%
10. Diabetes unspecified type	6444	3.14%	1.22%
11. Senility	5939	2.90%	1.13%
12. Unspecified GI Cancer	4087	1.99%	0.78%
13. Pulmonary Embolism	3750	1.83%	0.71%
14. Unspecified Heart Diseases	3471	1.69%	0.66%
15. Acute kidney failure	3311	1.62%	0.63%

Package Description 2016	Deaths	Percentage over total GCs	Percentage over total deaths
1. Other Ill Defined code for causes of death	34052	15.51%	5.88%
2. Shock, Cardiac Arrest, Coma	21562	9.82%	3.72%
3. Heart failure unspecified right or left	16608	7.56%	2.87%
4. Unspecified type of Stroke	15020	6.84%	2.59%
5. Unspecified lower respiratory infection	13649	6.22%	2.36%
6. Exposure to unspecified factor X59	9945	4.53%	1.72%
7. Unspecified Site Cancer	8884	4.05%	1.53%
8. Diabetes unspecified type	6978	3.18%	1.20%
9. Cardiac rhythm disorders	6593	3.00%	1.14%
10. Sepsis (Non- maternal and neonatal sepsis)	5275	2.40%	0.91%
11. Hypertension	4249	1.94%	0.73%
12. Left Heart Failure	4213	1.92%	0.73%
13. Senility	3911	1.78%	0.67%
14. Unspecified Heart Diseases	3470	1.58%	0.60%
15. Pneumonitis	3280	1.49%	0.57%

Class 1	Class 2
Class 3	Class 4

Supplementary Figure 2c. Ranking of Garbage Codes for Germany, both sexes combined, all ages, in number of deaths and percentage over the total of Garbage Codes and over total deaths per year, years 1990 and 2017.

Package Description 1990	Deaths	Percentage over total GCs	Percentage over total deaths
1. Unspecified type of Stroke	62384	20.89%	6.76%
2. Left Heart Failure	35189	11.79%	3.82%
3. Atherosclerosis	33795	11.32%	3.66%
4. Cardiac Diseases other	21221	7.11%	2.30%
5. Diabetes unspecified type	19473	6.52%	2.11%
6. Unspecified site Cancer	16868	5.65%	1.83%
7. Unspecified sign & symptom for Heart diseases	13243	4.44%	1.44%
8. Flutter and Fibrillation	10979	3.68%	1.19%
9. Arritmia	9195	3.08%	1.00%
10. Exposure to unspecified factor X59	7024	2.35%	0.76%
11. Hypertension	6621	2.22%	0.72%
12. Kidney Cancer	6100	2.04%	0.66%
13. Pulmonary Embolism	5383	1.80%	0.58%
14. Senility	4759	1.59%	0.52%
15. CKD	4196	1.41%	0.45%

Package Description 2017	Deaths	Percentage over total GCs	Percentage over total deaths
1. Heart failure unspecified right or left	31930	13.34%	3.43%
2. Other Ill Defined code for causes of death	25910	10.82%	2.78%
3. Unspecified type of Stroke	24027	10.04%	2.58%
4. Unspecified lower respiratory infection	18347	7.66%	1.97%
5. Diabetes unspecified type	12543	5.24%	1.35%
6. Unspecified Site Cancer	11930	4.98%	1.28%
7. Sepsis (Non- maternal and neonatal sepsis)	7764	3.24%	0.83%
8. Acute kidney failure	7403	3.09%	0.79%
9. Hypertension	7186	3.00%	0.77%
10. Left Heart Failure	7157	2.99%	0.77%
11. Unspecified Cardiomyopathy	6318	2.64%	0.68%
12. Pulmonary Embolism	5342	2.23%	0.57%
13. Cardiac rhythm disorders	5064	2.12%	0.54%
14. Non-follicular lymphoma, unspecified	4848	2.03%	0.52%
15. Unspecified Urinary Cancer	4101	1.71%	0.44%

Class 1	Class 2
Class 3	Class 4

Supplementary Figure 2d. Ranking of Garbage Codes for Greece, both sexes combined, all ages, in number of deaths and percentage over the total of Garbage Codes and over total deaths per year, years 1990 and 2016.

Package Description 1990	Deaths	Percentage over total GCs	Percentage over total deaths
1. Unspecified type of Stroke	15049	30.25%	15.98%
2. Shock, Cardiac Arrest, Coma	8642	17.37%	9.18%
3. Heart failure unspecified right or left	5013	10.08%	5.33%
4. Acute Respiratory Failure	2525	5.08%	2.68%
5. Left Heart Failure	2520	5.07%	2.68%
6. Senility	2097	4.22%	2.23%
7. Primary or secondary Liver Cancer Unspecified	1838	3.70%	1.95%
8. Acute kidney failure	1195	2.40%	1.27%
9. Unspecified site Cancer	1192	2.40%	1.27%
10. Motor vehicle accident of unspecified nature	942	1.89%	1.00%
11. Diabetes unspecified type	936	1.88%	0.99%
12. Pulmonary Embolism	704	1.42%	0.75%
13. Unspecified sign & symptom for Respiratory	627	1.26%	0.67%
14. Unspecified upper respiratory infection	624	1.25%	0.66%
15. Unspecified lower respiratory infection	609	1.22%	0.65%

Package Description 2016	Deaths	Percentage over total GCs	Percentage over total deaths
1. Unspecified type of Stroke	10011	20.79%	8.42%
2. Heart failure unspecified right or left	6921	14.37%	5.82%
3. Unspecified lower respiratory infection	5548	11.52%	4.66%
4. Shock, Cardiac Arrest, Coma	5409	11.23%	4.55%
5. Sepsis (Non- maternal and neonatal sepsis)	3624	7.53%	3.05%
6. Left Heart Failure	1989	4.13%	1.67%
7. Unspecified site Cancer	1756	3.65%	1.48%
8. Senility	1337	2.78%	1.12%
9. Pneumonitis	1200	2.49%	1.01%
10. Diabetes unspecified type	1051	2.18%	0.88%
11. Hypertension	964	2.00%	0.81%
12. Primary or secondary Liver Cancer Unspecified	952	1.98%	0.80%
13. Acute kidney failure	841	1.75%	0.71%
14. CKD due to diabetes Unspecified type	527	1.09%	0.44%
15. Pulmonary Embolism	526	1.09%	0.44%

Class 1	Class 2
Class 3	Class 4

Supplementary Figure 2e. Ranking of Garbage Codes for the Netherlands, both sexes combined, all ages, in number of deaths and percentage over the total of Garbage Codes and over total deaths per year, years 1990 and 2017.

Package Description 1990	Deaths	Percentage over total GCs	Percentage over total deaths
1. Unspecified type of Stroke	8312	20.36%	6.45%
2. Diabetes unspecified type	3600	8.82%	2.79%
3. Other Ill Defined code for causes of death	3565	8.73%	2.77%
4. Heart failure unspecified right or left	3242	7.94%	2.52%
5. Unspecified lower respiratory infection	2860	7.01%	2.22%
6. Unspecified site Cancer	2464	6.03%	1.91%
7. Unspecified Heart Diseases	1718	4.21%	1.33%
8. Unspecified Cardiomyopathy	1447	3.54%	1.12%
9. Shock, Cardiac Arrest, Coma	1255	3.07%	0.97%
10. Exposure to unspecified factor X59	1178	2.88%	0.91%
11. Left Heart Failure	1167	2.86%	0.91%
12. Atherosclerosis	971	2.38%	0.75%
13. Senility	875	2.14%	0.68%
14. Gastrointestinal Bleeding	786	1.93%	0.61%
15. Acute kidney failure	720	1.76%	0.56%

Package Description 2017	Deaths	Percentage over total GCs	Percentage over total deaths
1. Heart failure unspecified right or left	6639	12.81%	4.42%
2. Unspecified type of Stroke	5285	10.20%	3.52%
3. Other Ill Defined code for causes of death	4951	9.55%	3.30%
4. Unspecified lower respiratory infection	3812	7.35%	2.54%
5. Unspecified site Cancer	2264	4.37%	1.51%
6. Shock, Cardiac Arrest, Coma	2120	4.09%	1.41%
7. Diabetes unspecified type	1777	3.43%	1.18%
8. Senility	1734	3.35%	1.15%
9. Sepsis (Non- maternal and neonatal sepsis)	1607	3.10%	1.07%
10. Left Heart Failure	1249	2.41%	0.83%
11. Unspecified GI Cancer	1197	2.31%	0.80%
12. Non-follicular lymphoma, unspecified	1001	1.93%	0.67%
13. Gastrointestinal Bleeding	811	1.56%	0.54%
14. Acute kidney failure	786	1.52%	0.52%
15. Cardiac rhythm disorders	776	1.50%	0.52%

Class 1	Class 2
Class 3	Class 4

Supplementary Figure 2f. Ranking of Garbage Codes for Portugal, both sexes combined, all ages, in number of deaths and percentage over the total of Garbage Codes and over total deaths per year, years 1990 and 2017.

Package Description 1990	Deaths	Percentage over total GCs	Percentage over total deaths
1. Unspecified type of Stroke	20846	36.95%	20.22%
2. Senility	5626	9.97%	5.46%
3. Other Ill Defined code for causes of death	3978	7.05%	3.86%
4. Diabetes unspecified type	2737	4.85%	2.65%
5. Heart failure unspecified right or left	2707	4.80%	2.63%
6. Motor vehicle accident of unspecified nature	2695	4.78%	2.61%
7. Unspecified lower respiratory infection	2499	4.43%	2.42%
8. Atherosclerosis	2355	4.17%	2.28%
9. Left Heart Failure	1902	3.37%	1.84%
10. Shock, Cardiac Arrest, Coma	1170	2.07%	1.13%
11. Pulmonary Embolism	771	1.37%	0.75%
12. Unspecified site Cancer	662	1.17%	0.64%
13. Acute Respiratory Failure	589	1.04%	0.57%
14. Exposure to unspecified factor X59	471	0.83%	0.46%
15. Unspecified Heart Diseases	456	0.81%	0.44%

Package Description 2017	Deaths	Percentage over total GCs	Percentage over total deaths
1. Unspecified type of Stroke	8740	20.55%	7.93%
2. Unspecified lower respiratory infection	5352	12.58%	4.86%
3. Heart failure unspecified right or left	3994	9.39%	3.62%
4. Other Ill Defined code for causes of death	2936	6.90%	2.66%
5. Unspecified chronic respiratory diseases	1781	4.19%	1.62%
6. Diabetes unspecified type	1734	4.08%	1.57%
7. Shock, Cardiac Arrest, Coma	1641	3.86%	1.49%
8. Senility	1320	3.10%	1.20%
9. Unspecified site Cancer	1177	2.77%	1.07%
10. Left Heart Failure	1131	2.66%	1.03%
11. Sepsis (Non- maternal and neonatal sepsis)	1009	2.37%	0.92%
12. Hypertension	766	1.80%	0.70%
13. Exposure to unspecified factor X59	724	1.70%	0.66%
14. Pulmonary Embolism	663	1.56%	0.60%
15. Pneumonitis	600	1.41%	0.54%

Class 1	Class 2
Class 3	Class 4

Supplementary Figure 2g. Ranking of Garbage Codes for Spain, both sexes combined, all ages, in number of deaths and percentage over the total of Garbage Codes and over total deaths per year, years 1990 and 2017.

Package Description 1990	Deaths	Percentage over total GCs	Percentage over total deaths
1. Unspecified type of Stroke	22181	16.86%	6.66%
2. Heart failure unspecified right or left	15317	11.64%	4.60%
3. Atherosclerosis	8748	6.65%	2.63%
4. Diabetes unspecified type	8720	6.63%	2.62%
5. Unspecified lower respiratory infection	6098	4.63%	1.83%
6. Left Heart Failure	5702	4.33%	1.71%
7. Shock, Cardiac Arrest, Coma	5444	4.14%	1.63%
8. Unspecified site Cancer	5437	4.13%	1.63%
9. Motor vehicle accident of unspecified nature	5243	3.98%	1.57%
10. Acute kidney failure	3318	2.52%	1.00%
11. Acute Respiratory Failure	3300	2.51%	0.99%
12. Exposure to unspecified factor X59	2963	2.25%	0.89%
13. Unspecified Cardiomyopathy	2754	2.09%	0.83%
14. Unspecified Heart Diseases	2537	1.93%	0.76%
15. Primary or secondary Liver Cancer Unspecified	2402	1.83%	0.72%

Package Description 2017	Deaths	Percentage over total GCs	Percentage over total deaths
1. Heart failure unspecified right or left	16997	12.99%	4.00%
2. Unspecified chronic respiratory diseases	13884	10.61%	3.27%
3. Unspecified lower respiratory infection	10973	8.39%	2.58%
4. Unspecified type of Stroke	10732	8.20%	2.53%
5. Other Ill Defined code for causes of death	9349	7.15%	2.20%
6. Diabetes unspecified type	6559	5.01%	1.54%
7. Unspecified site Cancer	4560	3.49%	1.07%
8. Left Heart Failure	4310	3.29%	1.02%
9. Sepsis (Non- maternal and neonatal sepsis)	3988	3.05%	0.94%
10. Hypertension	3608	2.76%	0.85%
11. Acute kidney failure	2975	2.27%	0.70%
12. Unspecified Cardiomyopathy	2732	2.09%	0.64%
13. Cardiac rhythm disorders	2292	1.75%	0.54%
14. Pneumonitis	2155	1.65%	0.51%
15. Gastrointestinal Bleeding	2152	1.64%	0.51%

Class 1	Class 2
Class 3	Class 4

Supplementary Figure 2h. Ranking of Garbage Codes for Sweden, both sexes combined, all ages, in number of deaths and percentage over the total of Garbage Codes and over total deaths per year, years 1990 and 2017.

Package Description 1990	Deaths	Percentage over total GCs	Percentage over total deaths
1. Unspecified type of Stroke	5544	22.30%	5.84%
2. Unspecified lower respiratory infection	3636	14.63%	3.83%
3. Atherosclerosis	2013	8.10%	2.12%
4. Heart failure unspecified right or left	1739	7.00%	1.83%
5. Diabetes unspecified type	1476	5.94%	1.55%
6. Unspecified Heart Diseases	1238	4.98%	1.30%
7. Pulmonary Embolism	888	3.57%	0.94%
8. Left Heart Failure	882	3.55%	0.93%
9. Exposure to unspecified factor X59	848	3.41%	0.89%
10. Unspecified site Cancer	742	2.98%	0.78%
11. Senility	663	2.67%	0.70%
12. Other Ill Defined code for causes of death	514	2.07%	0.54%
13. Undetermined intent Poisoning by chemicals and substances	303	1.22%	0.32%
14. Primary or secondary Liver Cancer Unspecified	278	1.12%	0.29%
15. Unspecified Cardiomyopathy	275	1.11%	0.29%

Package Description 2017	Deaths	Percentage over total GCs	Percentage over total deaths
1. Heart failure unspecified right or left	3240	12.05%	3.52%
2. Unspecified type of Stroke	2944	10.95%	3.20%
3. Other Ill Defined code for causes of death	2031	7.56%	2.21%
4. Unspecified lower respiratory infection	1863	6.93%	2.02%
5. Diabetes unspecified type	1210	4.50%	1.31%
6. Unspecified site Cancer	1132	4.21%	1.23%
7. Exposure to unspecified factor X59	917	3.41%	1.00%
8. Senility	866	3.22%	0.94%
9. Sepsis (Non- maternal and neonatal sepsis)	832	3.09%	0.90%
10. Hypertension	761	2.83%	0.83%
11. Unspecified Infectious Diseases	622	2.31%	0.68%
12. Atherosclerosis	506	1.88%	0.55%
13. Undetermined intent Poisoning by multiple or unspecified drug	504	1.88%	0.55%
14. Pulmonary Embolism	396	1.47%	0.43%
15. Unspecified cardiovascular diseases	384	1.43%	0.42%

Class 1	Class 2
Class 3	Class 4

Supplementary Figure 2i. Ranking of Garbage Codes for the United Kingdom, both sexes combined, all ages, in number of deaths and percentage over the total of Garbage Codes and over total deaths per year, years 1990 and 2017.

Package Description 1990	Deaths	Percentage over total GCs	Percentage over total deaths
1. Unspecified type of Stroke	53947	31.85%	8.43%
2. Unspecified lower respiratory infection	27297	16.11%	4.27%
3. Unspecified site Cancer	14271	8.43%	2.23%
4. Diabetes unspecified type	8412	4.97%	1.31%
5. Heart failure unspecified right or left	6395	3.78%	1.00%
6. Other Ill Defined code for causes of death	5845	3.45%	0.91%
7. Unspecified Heart Diseases	4389	2.59%	0.69%
8. Atherosclerosis	4274	2.52%	0.67%
9. Non-rheumatic valvar unspecified	3088	1.82%	0.48%
10. Senility	2836	1.67%	0.44%
12. Unspecified urinary Cancer	2291	1.35%	0.36%
11. Acute kidney failure	2245	1.33%	0.35%
13. Unspecified cardiovascular diseases	2194	1.30%	0.34%
14. Unspecified Cardiomyopathy	1968	1.16%	0.31%
15. Other motor vehicle another motor vehicle	1869	1.10%	0.29%

Package Description 2017	Deaths	Percentage over total GCs	Percentage over total deaths
1. Unspecified lower respiratory infection	31660	19.84%	5.95%
2. Unspecified type of Stroke	19216	12.04%	3.61%
3. Unspecified site Cancer	8313	5.21%	1.56%
4. Senility	7722	4.84%	1.45%
5. Other Ill Defined code for causes of death	6681	4.19%	1.26%
6. Left Heart Failure	3747	2.35%	0.70%
7. Unspecified GI Cancer	3172	1.99%	0.60%
8. Alcoholic hepatic failure	3081	1.93%	0.58%
9. Undetermined intent Poisoning by multiple or unspecified drug	2988	1.87%	0.56%
10. Sepsis (Non- maternal and neonatal sepsis)	2742	1.72%	0.52%
11. Non-follicular lymphoma, unspecified	2708	1.70%	0.51%
12. Exposure to unspecified factor X59	2417	1.51%	0.45%
13. Pulmonary Embolism	2223	1.39%	0.42%
14. Diabetes unspecified type	2037	1.28%	0.38%
15. Unspecified Bronchitis and Bronchiectasis	1941	1.22%	0.36%

Class 1	Class 2
Class 3	Class 4

Supplementary Table 7. Percentage of Garbage code packages over total number of deaths, year 2017, both sexes, ranking on Italy.

In bold, the first ten for Italy. The other packages have been included to consider the first ten packages for all considered locations. On the right of each percentage column, the ranking for the specific location.

Package Description	Italy		Piemonte		Valle d'Aosta		Lombardia		Prov. Bolzano		Prov. Trento		Veneto		Friuli - Venezia Giulia		Liguria		Emilia - Romagna		Toscana		Umbria	
**Unspecified type of Stroke**	5.14%	1	6.32%	1	5.64%	1	4.55%	1	3.87%	1	2.67%	1	3.59%	1	4.78%	1	4.30%	1	3.91%	1	5.86%	1	4.77%	1
**Diabetes unspecified type**	2.26%	2	1.88%	3	1.24%	5	1.49%	3	1.16%	4	1.29%	6	1.91%	4	1.45%	3	2.10%	2	1.59%	5	1.86%	4	1.98%	2
**Unspecified Heart Diseases**	1.74%	3	1.67%	4	1.22%	6	1.46%	4	0.91%	7	1.62%	2	1.93%	3	1.34%	5	1.80%	3	1.62%	4	2.12%	2	1.45%	5
**Unspecified lower respiratory infectious**	1.68%	4	2.30%	2	2.53%	2	2.02%	2	1.52%	3	1.40%	3	2.27%	2	2.31%	2	1.79%	4	2.13%	2	2.10%	3	1.67%	3
**Exposure to unspecified factor X59**	1.34%	5	0.93%	8	0.35%	19	1.09%	7	0.09%	35	1.38%	4	1.03%	7	0.72%	11	1.37%	7	1.12%	7	1.20%	7	1.45%	4
**Sepsis (Non- maternal and neonatal sepsis)**	1.17%	6	1.37%	6	0.96%	8	1.21%	5	0.93%	6	1.17%	7	1.44%	5	1.38%	4	1.51%	6	1.90%	3	1.43%	6	1.44%	6
**Unspecified Cancer Site**	1.03%	7	1.15%	7	0.70%	11	1.19%	6	1.08%	5	1.31%	5	1.15%	6	1.16%	6	1.02%	8	1.13%	6	0.97%	8	0.93%	9
**Shock, Cardiac Arrest, Coma**	0.85%	8	0.48%	15	0.34%	21	0.58%	14	0.80%	9	0.55%	14	0.98%	8	0.50%	14	0.51%	16	0.40%	17	0.75%	12	0.65%	13
**Heart failure unspecified right or left**	0.84%	9	0.77%	10	0.42%	17	0.57%	15	2.46%	2	0.52%	15	0.73%	11	0.35%	18	0.82%	9	0.58%	10	0.79%	9	0.71%	11
**Senility**	0.84%	10	1.55%	5	1.57%	3	0.69%	11	0.31%	18	0.61%	11	0.53%	14	0.51%	12	0.53%	15	0.45%	15	1.82%	5	1.37%	7
Hypertension	0.72%	11	0.83%	9	0.79%	10	0.75%	9	0.57%	11	0.56%	13	0.74%	10	0.75%	9	0.64%	12	0.68%	9	0.78%	10	0.68%	12
Other ill-defined causes of death	0.71%	12	0.66%	11	0.97%	7	0.55%	16	0.55%	14	0.95%	9	0.75%	9	0.82%	8	1.52%	5	0.56%	11	0.77%	11	0.44%	16
Non-follicular lymphoma, unspecified	0.61%	13	0.59%	13	0.49%	14	0.73%	10	0.42%	16	0.82%	10	0.63%	12	0.50%	13	0.55%	13	0.68%	8	0.66%	14	0.77%	10
Primary or secondary Liver Cancer Unspecified	0.59%	14	0.39%	19	1.25%	4	0.63%	12	0.51%	15	1.00%	8	0.63%	13	0.73%	10	0.48%	17	0.39%	18	0.47%	18	0.45%	15
Left heart failure	0.59%	15	0.59%	14	0.47%	16	0.38%	19	0.83%	8	0.51%	16	0.42%	19	0.48%	16	0.76%	10	0.41%	16	0.61%	16	1.10%	8
Acute kidney failure	0.58%	16	0.45%	18	0.34%	20	0.50%	18	0.38%	17	0.42%	19	0.50%	17	0.24%	21	0.47%	18	0.49%	13	0.61%	17	0.47%	14
Unspecified cardiovascular diseases	0.57%	17	0.48%	16	0.21%	27	0.86%	8	0.20%	23	0.26%	22	0.28%	21	0.19%	26	0.70%	11	0.48%	14	0.66%	13	0.33%	19
Cardiac rhythm disorders	0.52%	18	0.60%	12	0.95%	9	0.58%	13	0.55%	13	0.45%	18	0.51%	15	0.49%	15	0.54%	14	0.51%	12	0.64%	15	0.25%	23
Unspecified Cardiomyopathy	0.46%	19	0.47%	17	0.30%	23	0.55%	17	0.62%	10	0.59%	12	0.46%	18	0.23%	22	0.45%	19	0.31%	20	0.39%	20	0.35%	17
Unspecified chronic respiratory diseases	0.26%	23	0.23%	24	0.27%	25	0.26%	23	0.16%	27	0.17%	28	0.19%	29	1.01%	7	0.20%	29	0.24%	22	0.22%	30	0.16%	33
Myocardial Degeneration	0.10%	41	0.02%	68	0.00%	72	0.04%	57	0.05%	48	0.00%	82	0.15%	35	0.19%	25	0.01%	87	0.04%	57	0.02%	63	0.04%	48

Package Description	Italy		Marche		Lazio		Abruzzo		Molise		Campania		Puglia		Basilicata		Calabria		Sardegna		Sicilia	
**Unspecified type of Stroke**	5.14%	1	4.09%	1	4.07%	1	4.83%	1	4.58%	1	7.20%	1	4.03%	1	4.78%	1	6.62%	1	4.35%	1	7.88%	1
**Diabetes unspecified type**	2.26%	2	1.59%	5	2.32%	2	2.50%	2	2.49%	2	3.69%	2	3.00%	2	3.05%	2	3.53%	2	1.73%	3	3.45%	2
**Unspecified Heart Diseases**	1.74%	3	1.95%	3	1.90%	4	1.71%	4	2.33%	3	1.82%	3	1.54%	3	1.48%	4	2.51%	3	1.63%	4	1.74%	4
**Unspecified lower respiratory infectious**	1.68%	4	2.13%	2	1.92%	3	1.30%	5	1.03%	7	0.65%	12	0.94%	8	0.83%	7	0.56%	17	1.39%	6	0.98%	8
**Exposure to unspecified factor X59**	1.34%	5	1.54%	6	1.84%	5	1.90%	3	1.89%	4	1.62%	4	1.40%	4	1.52%	3	1.86%	4	1.57%	5	1.63%	5
**Sepsis (Non- maternal and neonatal sepsis)**	1.17%	6	1.65%	4	0.96%	7	0.85%	8	0.66%	14	0.68%	11	1.01%	6	1.02%	5	0.96%	7	1.01%	8	0.56%	16
**Unspecified Cancer Site**	1.03%	7	1.01%	7	0.95%	8	0.98%	6	0.78%	12	0.81%	10	0.96%	7	0.76%	8	0.90%	8	1.29%	7	0.86%	9
**Shock, Cardiac Arrest, Coma**	0.85%	8	0.46%	14	0.70%	10	0.67%	9	1.15%	6	1.41%	5	1.01%	5	0.69%	10	1.47%	5	1.00%	9	1.80%	3
**Heart failure unspecified right or left**	0.84%	9	0.60%	9	1.10%	6	0.89%	7	1.03%	8	1.31%	7	0.82%	9	0.75%	9	1.14%	6	0.56%	14	1.11%	7
**Senility**	0.84%	10	0.39%	19	0.33%	20	0.46%	17	0.74%	13	0.31%	22	0.78%	10	0.54%	18	0.87%	10	1.80%	2	1.55%	6
Hypertension	0.72%	11	0.52%	11	0.68%	11	0.67%	10	0.90%	9	0.63%	13	0.69%	14	0.61%	14	0.81%	11	0.91%	10	0.79%	10
Other ill-defined causes of death	0.71%	12	0.46%	13	0.47%	17	0.49%	15	0.52%	16	1.35%	6	0.48%	19	0.64%	12	0.81%	12	0.67%	12	0.73%	11
Non-follicular lymphoma, unspecified	0.61%	13	0.53%	10	0.65%	12	0.55%	13	0.45%	19	0.62%	14	0.55%	17	0.44%	21	0.38%	21	0.67%	13	0.47%	19
Primary or secondary Liver Cancer Unspecified	0.59%	14	0.44%	16	0.49%	16	0.42%	18	0.55%	15	0.91%	8	0.72%	13	0.53%	19	0.65%	15	0.74%	11	0.69%	14
Left heart failure	0.59%	15	0.49%	12	0.85%	9	0.65%	11	0.80%	11	0.53%	17	0.67%	15	0.61%	13	0.89%	9	0.47%	16	0.72%	12
Acute kidney failure	0.58%	16	0.45%	15	0.64%	13	0.48%	16	0.82%	10	0.84%	9	0.76%	12	0.54%	17	0.72%	13	0.53%	15	0.71%	13
Unspecified cardiovascular diseases	0.57%	17	0.44%	17	0.51%	15	0.51%	14	0.39%	22	0.62%	15	0.60%	16	0.60%	15	0.67%	14	0.37%	18	0.64%	15
Cardiac rhythm disorders	0.52%	18	0.43%	18	0.42%	18	0.57%	12	0.49%	18	0.34%	20	0.77%	11	0.98%	6	0.53%	18	0.37%	19	0.41%	21
Unspecified Cardiomyopathy	0.46%	19	0.33%	22	0.52%	14	0.37%	19	0.51%	17	0.44%	18	0.39%	21	0.67%	11	0.57%	16	0.34%	20	0.55%	17
Unspecified chronic respiratory diseases	0.26%	23	0.18%	31	0.21%	29	0.22%	26	0.17%	33	0.24%	26	0.29%	23	0.34%	22	0.27%	25	0.21%	25	0.43%	20
Myocardial Degeneration	0.10%	41	0.82%	8	0.33%	19	0.26%	24	1.35%	5	0.01%	74	0.01%	72	0.02%	66	0.01%	69	0.00%	95	0.00%	90

Supplementary Table 8a. Piemonte 2017, deaths added during redistribution to the 15 most affected Level 4 underlying causes of death (overall deaths redistributed=10,265)

Level 4 underlying cause of death	Deaths before redistribution	Deaths after redistribution	Deaths added	Percent of overall redistributed	Cumulative percentage
Ischemic stroke	1107	4394	3287	32.02%	32.02%
Diabetes mellitus type 2	257	1379	1122	10.93%	42.95%
Chronic ischemic heart disease*	2664	3651	987	9.61%	52.56%
Intracerebral haemorrhage	859	1557	698	6.80%	59.36%
Influenza*	54	679	625	6.09%	65.45%
Acute myocardial infarction*	1879	2485	606	5.90%	71.35%
Pneumococcal pneumonia*	14	438	424	4.13%	75.49%
Non-Hodgkin lymphoma	122	501	379	3.69%	79.18%
Other cardiomyopathy	72	387	315	3.07%	82.25%
Other lower respiratory infections*	137	441	305	2.97%	85.22%
Chronic kidney disease due to hypertension	324	574	250	2.43%	87.65%
Chronic kidney disease due to diabetes mellitus type 2	29	168	139	1.35%	89.00%
Subarachnoid haemorrhage	183	298	115	1.12%	90.12%
Non-rheumatic calcific aortic valve disease	308	409	102	0.99%	91.11%
Chronic kidney disease due to other and unspecified causes	10	73	63	0.61%	91.72%

* These are a further subdivision of Level 4 causes, used for redistribution. In particular: “chronic ischemic heart disease” and “acute myocardial infarction” merge into Level 4 “ischemic heart disease”; “influenza”, “pneumococcal pneumonia” and “other lower respiratory infections” merge into Level 4 “lower respiratory infections”.

Supplementary Table 8b. Valle d'Aosta 2017, deaths added during redistribution to the 15 most affected Level 4 underlying causes of death (overall deaths redistributed=270)

Level 4 underlying cause of death	Deaths before redistribution	Deaths after redistribution	Deaths added	Percent of overall redistributed	Cumulative percentage
Ischemic stroke	24	112	88	32.64%	32.64%
Other lower respiratory infections*	5	35	30	11.21%	43.85%
Chronic ischemic heart disease*	70	95	25	9.30%	53.15%
Diabetes mellitus type 2	9	31	22	8.22%	61.37%
Acute myocardial infarction*	61	81	20	7.46%	68.83%
Intracerebral haemorrhage	20	38	18	6.72%	75.55%
Non-Hodgkin lymphoma	2	11	9	3.44%	78.99%
Other cardiomyopathy	3	9	6	2.27%	81.26%
Pneumococcal pneumonia*	0	6	6	2.21%	83.47%
Chronic kidney disease due to hypertension	10	15	6	2.04%	85.51%
Chronic kidney disease due to diabetes mellitus type 2	3	7	4	1.40%	86.91%
Influenza*	2	5	3	1.27%	88.18%
Non-rheumatic calcific aortic valve disease	11	14	3	1.03%	89.21%
Subarachnoid haemorrhage	1	4	3	1.01%	90.23%
Diverticular disease of intestines*	5	7	2	0.64%	90.86%

* These are a further subdivision of Level 4 causes, used for redistribution. In particular: "chronic ischemic heart disease" and "acute myocardial infarction" merge into Level 4 "ischemic heart disease"; "influenza", "pneumococcal pneumonia" and "other lower respiratory infections" merge into Level 4 "lower respiratory infections"; "diverticular disease of intestines" merges into "other digestive diseases".

Supplementary Table 8c. Lombardia 2017, deaths added during redistribution to the 15 most affected Level 4 underlying causes of death (overall deaths redistributed=16,341**)**

**Level 4 underlying cause of death**	**Deaths before redistribution**	**Deaths after redistribution**	**Deaths added**	**Percent of overall redistributed**	**Cumulative percentage**
Ischemic stroke	1422	5939	4517	27.64%	27.64%
Chronic ischemic heart disease*	5518	7217	1699	10.40%	38.04%
Diabetes mellitus type 2	401	2060	1659	10.15%	48.19%
Acute myocardial infarction*	3972	5108	1136	6.95%	55.14%
Intracerebral haemorrhage	1667	2723	1056	6.46%	61.60%
Influenza*	120	1141	1022	6.25%	67.85%
Pneumococcal pneumonia*	32	983	951	5.82%	73.67%
Non-Hodgkin lymphoma	224	1069	845	5.17%	78.85%
Other cardiomyopathy	134	818	684	4.19%	83.03%
Chronic kidney disease due to hypertension	738	1173	434	2.66%	85.69%
Chronic kidney disease due to diabetes mellitus type 2	81	357	277	1.69%	87.38%
Other lower respiratory infections*	163	415	252	1.54%	88.92%
Subarachnoid haemorrhage	324	500	176	1.08%	90.00%
Non-rheumatic calcific aortic valve disease	628	803	175	1.07%	91.07%
Pedestrian road injuries	101	200	99	0.61%	91.68%

* These are a further subdivision of Level 4 causes, used for redistribution. In particular: “chronic ischemic heart disease” and “acute myocardial infarction” merge into Level 4 “ischemic heart disease”; “influenza”, “pneumococcal pneumonia” and “other lower respiratory infections” merge into Level 4 “lower respiratory infections”.

Supplementary Table 8d. Provincia Autonoma di Bolzano 2017, deaths added during redistribution to the 15 most affected Level 4 underlying causes of death (overall deaths redistributed=643)

Level 4 underlying cause of death	Deaths before redistribution	Deaths after redistribution	Deaths added	Percent of overall redistributed	Cumulative percentage
Ischemic stroke	81	254	174	27.02%	27.02%
Chronic ischemic heart disease*	253	327	74	11.45%	38.46%
Diabetes mellitus type 2	18	80	61	9.56%	48.02%
Acute myocardial infarction*	219	272	53	8.18%	56.21%
Influenza*	18	60	42	6.55%	62.75%
Intracerebral haemorrhage	55	94	40	6.20%	68.95%
Other cardiomyopathy	3	39	36	5.61%	74.57%
Other lower respiratory infections*	8	33	25	3.90%	78.47%
Non-Hodgkin lymphoma	9	32	23	3.65%	82.11%
Chronic kidney disease due to hypertension	38	59	20	3.14%	85.26%
Non-rheumatic calcific aortic valve disease	49	60	11	1.72%	86.98%
Chronic kidney disease due to diabetes mellitus type 2	3	11	8	1.31%	88.29%
Subarachnoid haemorrhage	18	24	6	1.00%	89.29%
Opioid use disorders	0	5	5	0.84%	90.12%
Pneumococcal pneumonia*	1	6	5	0.81%	90.93%

* These are a further subdivision of Level 4 causes, used for redistribution. In particular: “chronic ischemic heart disease” and “acute myocardial infarction” merge into Level 4 “ischemic heart disease”; “influenza”, “pneumococcal pneumonia” and “other lower respiratory infections” merge into Level 4 “lower respiratory infections”.

Supplementary Table 8e. Provincia Autonoma di Trento 2017, deaths added during redistribution to the 15 most affected Level 4 underlying causes of death (overall deaths redistributed=701)

Level 4 underlying cause of death	Deaths before redistribution	Deaths after redistribution	Deaths added	Percent of overall redistributed	Cumulative percentage
Ischemic stroke	85	234	149	21.32%	21.32%
Chronic ischemic heart disease*	396	492	96	13.66%	34.98%
Diabetes mellitus type 2	23	98	75	10.64%	45.62%
Pneumococcal pneumonia*	5	57	52	7.44%	53.06%
Non-Hodgkin lymphoma	8	56	47	6.77%	59.84%
Acute myocardial infarction*	205	248	43	6.15%	65.99%
Intracerebral haemorrhage	77	111	34	4.89%	70.88%
Other cardiomyopathy	4	38	34	4.89%	75.76%
Other lower respiratory infections*	4	29	25	3.60%	79.37%
Chronic kidney disease due to hypertension	40	60	19	2.76%	82.13%
Chronic kidney disease due to diabetes mellitus type 2	0	11	11	1.61%	83.73%
Non-rheumatic calcific aortic valve disease	45	54	9	1.30%	85.04%
Self-harm by other specified means	49	56	8	1.08%	86.11%
Pedestrian road injuries	4	11	6	0.91%	87.03%
Subarachnoid haemorrhage	12	18	6	0.84%	87.87%

* These are a further subdivision of Level 4 causes, used for redistribution. In particular: “chronic ischemic heart disease” and “acute myocardial infarction” merge into Level 4 “ischemic heart disease”; “influenza”, “pneumococcal pneumonia” and “other lower respiratory infections” merge into Level 4 “lower respiratory infections”.

Supplementary Table 8f. Veneto 2017, deaths added during redistribution to the 15 most affected Level 4 underlying causes of death (overall deaths redistributed=8,299**)**

Level 4 underlying cause of death	Deaths before redistribution	Deaths after redistribution	Deaths added	Percent of overall redistributed	Cumulative percentage
Ischemic stroke	863	2776	1913	23.06%	23.06%
Diabetes mellitus type 2	209	1268	1059	12.77%	35.82%
Chronic ischemic heart disease*	3071	3992	921	11.10%	46.92%
Acute myocardial infarction*	1939	2486	547	6.59%	53.51%
Influenza*	53	555	502	6.05%	59.56%
Intracerebral haemorrhage	792	1249	458	5.52%	65.08%
Pneumococcal pneumonia*	9	443	434	5.23%	70.31%
Non-Hodgkin lymphoma	102	480	378	4.56%	74.87%
Other cardiomyopathy	69	403	335	4.03%	78.90%
Other lower respiratory infections*	77	389	312	3.76%	82.66%
Chronic kidney disease due to hypertension	407	647	240	2.89%	85.55%
Chronic kidney disease due to diabetes mellitus type 2	37	192	155	1.87%	87.43%
Non-rheumatic calcific aortic valve disease	535	655	120	1.44%	88.87%
Subarachnoid haemorrhage	116	189	73	0.88%	89.75%
Pedestrian road injuries	52	109	57	0.69%	90.44%

* These are a further subdivision of Level 4 causes, used for redistribution. In particular: “chronic ischemic heart disease” and “acute myocardial infarction” merge into Level 4 “ischemic heart disease”; “influenza”, “pneumococcal pneumonia” and “other lower respiratory infections” merge into Level 4 “lower respiratory infections”.

Supplementary Table 8g. Friuli – Venezia Giulia 2017, deaths added during redistribution to the 15 most affected Level 4 underlying causes of death (overall deaths redistributed=2,289)

Level 4 underlying cause of death	Deaths before redistribution	Deaths after redistribution	Deaths added	Percent of overall redistributed	Cumulative percentage
Ischemic stroke	275	966	691	30.21%	30.21%
Diabetes mellitus type 2	90	327	237	10.38%	40.58%
Chronic ischemic heart disease*	976	1181	205	8.97%	49.55%
Pneumococcal pneumonia*	7	194	187	8.16%	57.71%
Intracerebral haemorrhage	227	378	151	6.58%	64.29%
Acute myocardial infarction*	626	760	134	5.87%	70.16%
Influenza*	15	148	134	5.84%	75.99%
Non-Hodgkin lymphoma	30	120	90	3.91%	79.91%
Chronic kidney disease due to hypertension	142	204	62	2.72%	82.63%
Other cardiomyopathy	8	67	58	2.55%	85.18%
Other lower respiratory infections*	21	64	43	1.90%	87.08%
Subarachnoid haemorrhage	33	57	24	1.05%	88.13%
Non-rheumatic calcific aortic valve disease	123	145	22	0.95%	89.08%
Chronic kidney disease due to diabetes mellitus type 2	11	32	21	0.92%	90.01%
Myocarditis	23	43	20	0.86%	90.86%

* These are a further subdivision of Level 4 causes, used for redistribution. In particular: “chronic ischemic heart disease” and “acute myocardial infarction” merge into Level 4 “ischemic heart disease”; “influenza”, “pneumococcal pneumonia” and “other lower respiratory infections” merge into Level 4 “lower respiratory infections”.

Supplementary Table 8h. Liguria 2017, deaths added during redistribution to the 15 most affected Level 4 underlying causes of death (overall deaths redistributed=3,814**)**

Level 4 underlying cause of death	Deaths before redistribution	Deaths after redistribution	Deaths added	Percent of overall redistributed	Cumulative percentage
Ischemic stroke	537	1514	977	25.62%	25.62%
Diabetes mellitus type 2	104	624	520	13.63%	39.25%
Chronic ischemic heart disease*	1340	1796	455	11.94%	51.19%
Acute myocardial infarction*	751	995	243	6.38%	57.57%
Intracerebral haemorrhage	359	588	229	6.00%	63.57%
Influenza*	27	200	174	4.55%	68.13%
Non-Hodgkin lymphoma	32	183	151	3.97%	72.10%
Pneumococcal pneumonia*	6	153	148	3.87%	75.96%
Other cardiomyopathy	9	137	127	3.34%	79.30%
Other lower respiratory infections*	39	161	122	3.19%	82.49%
Chronic kidney disease due to hypertension	185	298	113	2.97%	85.47%
Chronic kidney disease due to diabetes mellitus type 2	13	78	65	1.71%	87.17%
Non-rheumatic calcific aortic valve disease	145	192	47	1.22%	88.40%
Subarachnoid haemorrhage	63	100	37	0.98%	89.37%
Pedestrian road injuries	18	45	27	0.71%	90.08%

* These are a further subdivision of Level 4 causes, used for redistribution. In particular: “chronic ischemic heart disease” and “acute myocardial infarction” merge into Level 4 “ischemic heart disease”; “influenza”, “pneumococcal pneumonia” and “other lower respiratory infections” merge into Level 4 “lower respiratory infections”.

Supplementary Table 8i. Emilia-Romagna 2017, deaths added during redistribution to the 15 most affected Level 4 underlying causes of death (overall deaths redistributed=7,978)

Level 4 underlying cause of death	Deaths before redistribution	Deaths after redistribution	Deaths added	Percent of overall redistributed	Cumulative percentage
Ischemic stroke	846	2892	2046	25.64%	25.64%
Diabetes mellitus type 2	294	1220	926	11.61%	37.25%
Chronic ischemic heart disease*	2964	3769	805	10.09%	47.34%
Pneumococcal pneumonia*	26	595	569	7.14%	54.48%
Influenza*	70	594	524	6.57%	61.05%
Acute myocardial infarction*	1944	2436	492	6.17%	67.22%
Intracerebral haemorrhage	881	1341	460	5.77%	72.99%
Non-Hodgkin lymphoma	109	535	426	5.34%	78.33%
Chronic kidney disease due to hypertension	417	652	235	2.94%	81.27%
Other cardiomyopathy	54	276	222	2.79%	84.06%
Chronic kidney disease due to diabetes mellitus type 2	47	176	129	1.62%	85.68%
Other lower respiratory infections*	93	222	129	1.62%	87.29%
Non-rheumatic calcific aortic valve disease	416	511	95	1.19%	88.48%
Subarachnoid haemorrhage	134	208	75	0.94%	89.42%
Urinary tract infections and interstitial nephritis	277	348	71	0.89%	90.31%

* These are a further subdivision of Level 4 causes, used for redistribution. In particular: “chronic ischemic heart disease” and “acute myocardial infarction” merge into Level 4 “ischemic heart disease”; “influenza”, “pneumococcal pneumonia” and “other lower respiratory infections” merge into Level 4 “lower respiratory infections”.

Supplementary Table 8j. Toscana 2017, deaths added during redistribution to the 15 most affected Level 4 underlying causes of death (overall deaths redistributed=8,964)

Level 4 underlying cause of death	Deaths before redistribution	Deaths after redistribution	Deaths added	Percent of overall redistributed	Cumulative percentage
Ischemic stroke	808	3496	2688	29.99%	29.99%
Chronic ischemic heart disease*	2569	3763	1194	13.32%	43.30%
Diabetes mellitus type 2	240	1194	954	10.64%	53.94%
Intracerebral haemorrhage	913	1497	583	6.51%	60.45%
Pneumococcal pneumonia*	16	561	545	6.08%	66.53%
Acute myocardial infarction*	1344	1853	509	5.68%	72.21%
Non-Hodgkin lymphoma	57	416	359	4.00%	76.21%
Influenza*	42	367	325	3.63%	79.84%
Other cardiomyopathy	27	269	242	2.70%	82.54%
Chronic kidney disease due to hypertension	241	467	226	2.52%	85.06%
Other lower respiratory infections*	56	252	195	2.18%	87.24%
Chronic kidney disease due to diabetes mellitus type 2	39	165	126	1.40%	88.64%
Non-rheumatic calcific aortic valve disease	283	399	117	1.30%	89.95%
Subarachnoid haemorrhage	134	223	89	1.00%	90.94%
Pedestrian road injuries	70	120	50	0.55%	91.50%

* These are a further subdivision of Level 4 causes, used for redistribution. In particular: “chronic ischemic heart disease” and “acute myocardial infarction” merge into Level 4 “ischemic heart disease”; “influenza”, “pneumococcal pneumonia” and “other lower respiratory infections” merge into Level 4 “lower respiratory infections”.

Supplementary Table 8k. Umbria 2017, deaths added during redistribution to the 15 most affected Level 4 underlying causes of death (overall deaths redistributed=1,873)

Level 4 underlying cause of death	Deaths before redistribution	Deaths after redistribution	Deaths added	Percent of overall redistributed	Cumulative percentage
Ischemic stroke	209	729	520	27.76%	27.76%
Chronic ischemic heart disease*	927	1175	248	13.23%	40.99%
Diabetes mellitus type 2	48	287	239	12.75%	53.74%
Intracerebral haemorrhage	162	273	111	5.91%	59.65%
Acute myocardial infarction*	410	507	97	5.20%	64.85%
Non-Hodgkin lymphoma	8	103	95	5.08%	69.93%
Other lower respiratory infections*	10	95	85	4.54%	74.47%
Pneumococcal pneumonia*	5	67	61	3.26%	77.73%
Influenza*	18	75	57	3.06%	80.79%
Chronic kidney disease due to hypertension	78	130	53	2.83%	83.62%
Other cardiomyopathy	10	62	52	2.79%	86.41%
Chronic kidney disease due to diabetes mellitus type 2	9	39	30	1.59%	88.00%
Subarachnoid haemorrhage	26	45	19	1.00%	88.99%
Non-rheumatic calcific aortic valve disease	52	70	18	0.95%	89.95%
Pedestrian road injuries	11	25	14	0.73%	90.68%

* These are a further subdivision of Level 4 causes, used for redistribution. In particular: “chronic ischemic heart disease” and “acute myocardial infarction” merge into Level 4 “ischemic heart disease”; “influenza”, “pneumococcal pneumonia” and “other lower respiratory infections” merge into Level 4 “lower respiratory infections”.

Supplementary Table 8l. Marche 2017, deaths added during redistribution to the 15 most affected Level 4 underlying causes of death (overall deaths redistributed=3,065)

Level 4 underlying cause of death	Deaths before redistribution	Deaths after redistribution	Deaths added	Percent of overall redistributed	Cumulative percentage
Ischemic stroke	459	1233	774	25.26%	25.26%
Chronic ischemic heart disease*	1500	1873	373	12.17%	37.43%
Diabetes mellitus type 2	103	438	335	10.93%	48.36%
Other lower respiratory infections*	18	236	218	7.10%	55.46%
Other cardiomyopathy	21	228	207	6.76%	62.22%
Influenza*	17	198	181	5.92%	68.14%
Intracerebral haemorrhage	338	503	165	5.38%	73.52%
Acute myocardial infarction*	661	812	151	4.91%	78.43%
Non-Hodgkin lymphoma	22	140	118	3.85%	82.28%
Chronic kidney disease due to hypertension	153	233	80	2.60%	84.88%
Chronic kidney disease due to diabetes mellitus type 2	16	53	37	1.22%	86.10%
Non-rheumatic calcific aortic valve disease	125	156	31	1.00%	87.10%
Pneumococcal pneumonia*	0	28	28	0.91%	88.01%
Myocarditis	10	38	28	0.91%	88.92%
Subarachnoid haemorrhage	51	78	27	0.88%	89.80%

* These are a further subdivision of Level 4 causes, used for redistribution. In particular: “chronic ischemic heart disease” and “acute myocardial infarction” merge into Level 4 “ischemic heart disease”; “influenza”, “pneumococcal pneumonia” and “other lower respiratory infections” merge into Level 4 “lower respiratory infections”.

Supplementary Table 8m. Lazio 2017, deaths added during redistribution to the 15 most affected Level 4 underlying causes of death (overall deaths redistributed=10,561)

Level 4 underlying cause of death	Deaths before redistribution	Deaths after redistribution	Deaths added	Percent of overall redistributed	Cumulative percentage
Ischemic stroke	792	3198	2406	22.78%	22.78%
Diabetes mellitus type 2	278	1811	1533	14.51%	37.30%
Chronic ischemic heart disease*	4783	6124	1341	12.70%	50.00%
Influenza*	57	724	667	6.32%	56.31%
Intracerebral haemorrhage	977	1592	615	5.83%	62.14%
Acute myocardial infarction*	2157	2736	579	5.49%	67.63%
Other cardiomyopathy	43	605	563	5.33%	72.95%
Non-Hodgkin lymphoma	62	526	464	4.39%	77.35%
Other lower respiratory infections*	61	406	345	3.26%	80.61%
Pneumococcal pneumonia*	10	302	293	2.77%	83.38%
Chronic kidney disease due to hypertension	401	677	276	2.62%	86.00%
Chronic kidney disease due to diabetes mellitus type 2	41	215	173	1.64%	87.64%
Non-rheumatic calcific aortic valve disease	327	436	109	1.03%	88.68%
Subarachnoid haemorrhage	175	276	101	0.96%	89.63%
Pedestrian road injuries	102	197	95	0.90%	90.53%

* These are a further subdivision of Level 4 causes, used for redistribution. In particular: “chronic ischemic heart disease” and “acute myocardial infarction” merge into Level 4 “ischemic heart disease”; “influenza”, “pneumococcal pneumonia” and “other lower respiratory infections” merge into Level 4 “lower respiratory infections”.

Supplementary Table 8n. Abruzzo 2017, deaths added during redistribution to the 15 most affected Level 4 underlying causes of death (overall deaths redistributed=2,673)

Level 4 underlying cause of death	Deaths before redistribution	Deaths after redistribution	Deaths added	Percent of overall redistributed	Cumulative percentage
Ischemic stroke	296	1020	724	27.10%	27.10%
Diabetes mellitus type 2	54	471	417	15.58%	42.68%
Chronic ischemic heart disease*	1403	1740	337	12.59%	55.28%
Intracerebral haemorrhage	251	418	167	6.26%	61.54%
Acute myocardial infarction*	601	734	133	4.98%	66.52%
Other lower respiratory infections*	18	150	132	4.95%	71.47%
Other cardiomyopathy	18	123	105	3.92%	75.39%
Non-Hodgkin lymphoma	12	114	102	3.80%	79.19%
Chronic kidney disease due to hypertension	177	256	79	2.97%	82.17%
Influenza*	13	77	65	2.42%	84.58%
Chronic kidney disease due to diabetes mellitus type 2	13	54	41	1.53%	86.12%
Non-rheumatic calcific aortic valve disease	125	154	29	1.09%	87.20%
Subarachnoid haemorrhage	55	83	28	1.04%	88.25%
Pedestrian road injuries	19	46	27	1.00%	89.25%
Pneumococcal pneumonia*	0	24	24	0.90%	90.15%

* These are a further subdivision of Level 4 causes, used for redistribution. In particular: “chronic ischemic heart disease” and “acute myocardial infarction” merge into Level 4 “ischemic heart disease”; “influenza”, “pneumococcal pneumonia” and “other lower respiratory infections” merge into Level 4 “lower respiratory infections”.

Supplementary Table 8o. Molise 2017, deaths added during redistribution to the 15 most affected Level 4 underlying causes of death (overall deaths redistributed=716)

Level 4 underlying cause of death	Deaths before redistribution	Deaths after redistribution	Deaths added	Percent of overall redistributed	Cumulative percentage
Ischemic stroke	60	236	175	24.50%	24.50%
Diabetes mellitus type 2	17	121	104	14.51%	39.00%
Chronic ischemic heart disease*	364	467	103	14.44%	53.44%
Other cardiomyopathy	0	71	70	9.84%	63.29%
Intracerebral haemorrhage	75	118	43	6.03%	69.32%
Acute myocardial infarction*	150	191	41	5.66%	74.98%
Other lower respiratory infections*	4	39	35	4.89%	79.87%
Non-Hodgkin lymphoma	3	24	21	2.97%	82.84%
Chronic kidney disease due to hypertension	38	58	20	2.73%	85.57%
Chronic kidney disease due to diabetes mellitus type 2	0	8	8	1.15%	86.72%
Non-rheumatic calcific aortic valve disease	19	26	7	0.98%	87.71%
Myocarditis	1	8	7	0.94%	88.64%
Subarachnoid haemorrhage	6	13	6	0.90%	89.54%
Pneumococcal pneumonia*	0	6	6	0.84%	90.38%
Pedestrian road injuries	5	11	6	0.79%	91.17%

* These are a further subdivision of Level 4 causes, used for redistribution. In particular: “chronic ischemic heart disease” and “acute myocardial infarction” merge into Level 4 “ischemic heart disease”; “influenza”, “pneumococcal pneumonia” and “other lower respiratory infections” merge into Level 4 “lower respiratory infections”.

Supplementary Table 8p. Campania 2017, deaths added during redistribution to the 15 most affected Level 4 underlying causes of death (overall deaths redistributed=11,593)

Level 4 underlying cause of death	Deaths before redistribution	Deaths after redistribution	Deaths added	Percent of overall redistributed	Cumulative percentage
Ischemic stroke	567	4183	3616	31.19%	31.19%
Diabetes mellitus type 2	385	2587	2202	19.00%	50.18%
Chronic ischemic heart disease*	4644	5909	1265	10.91%	61.10%
Intracerebral haemorrhage	794	1678	884	7.63%	68.73%
Acute myocardial infarction*	2229	2857	628	5.42%	74.14%
Non-Hodgkin lymphoma	23	423	400	3.45%	77.60%
Other cardiomyopathy	29	357	328	2.83%	80.43%
Chronic kidney disease due to diabetes mellitus type 2	66	362	296	2.56%	82.99%
Other lower respiratory infections*	58	328	270	2.33%	85.32%
Chronic kidney disease due to hypertension	604	840	237	2.04%	87.36%
Influenza*	40	208	168	1.45%	88.81%
Subarachnoid haemorrhage	132	286	154	1.33%	90.14%
Non-rheumatic calcific aortic valve disease	223	314	91	0.79%	90.92%
Pedestrian road injuries	29	108	79	0.68%	91.61%
Motor vehicle road injuries	72	144	72	0.62%	92.22%

* These are a further subdivision of Level 4 causes, used for redistribution. In particular: "chronic ischemic heart disease" and "acute myocardial infarction" merge into Level 4 "ischemic heart disease"; "influenza", "pneumococcal pneumonia" and "other lower respiratory infections" merge into Level 4 "lower respiratory infections".

Supplementary Table 8q. Puglia 2017, deaths added during redistribution to the 15 most affected Level 4 underlying causes of death (overall deaths redistributed=6,756)

Level 4 underlying cause of death	Deaths before redistribution	Deaths after redistribution	Deaths added	Percent of overall redistributed	Cumulative percentage
Ischemic stroke	542	2157	1616	23.91%	23.91%
Diabetes mellitus type 2	228	1532	1304	19.31%	43.22%
Chronic ischemic heart disease*	2909	3768	858	12.71%	55.93%
Intracerebral haemorrhage	582	1003	421	6.23%	62.15%
Acute myocardial infarction*	1295	1664	369	5.47%	67.62%
Non-Hodgkin lymphoma	37	311	273	4.05%	71.67%
Other lower respiratory infections*	37	291	254	3.76%	75.43%
Chronic kidney disease due to hypertension	593	805	212	3.13%	78.56%
Other cardiomyopathy	22	229	207	3.07%	81.63%
Chronic kidney disease due to diabetes mellitus type 2	51	238	187	2.77%	84.40%
Influenza*	16	180	164	2.43%	86.83%
Chronic kidney disease due to diabetes mellitus type 1	22	93	71	1.06%	87.89%
Subarachnoid haemorrhage	75	146	70	1.04%	88.93%
Non-rheumatic calcific aortic valve disease	207	273	67	0.98%	89.91%
Pneumococcal pneumonia*	1	57	56	0.83%	90.75%

* These are a further subdivision of Level 4 causes, used for redistribution. In particular: “chronic ischemic heart disease” and “acute myocardial infarction” merge into Level 4 “ischemic heart disease”; “influenza”, “pneumococcal pneumonia” and “other lower respiratory infections” merge into Level 4 “lower respiratory infections”.

Supplementary Table 8r. Basilicata 2017, deaths added during redistribution to the 15 most affected Level 4 underlying causes of death (overall deaths redistributed=1,123)

Level 4 underlying cause of death	Deaths before redistribution	Deaths after redistribution	Deaths added	Percent of overall redistributed	Cumulative percentage
Ischemic stroke	120	424	303	27.02%	27.02%
Diabetes mellitus type 2	25	234	209	18.58%	45.59%
Chronic ischemic heart disease*	434	556	122	10.85%	56.44%
Intracerebral haemorrhage	96	168	72	6.42%	62.86%
Acute myocardial infarction*	263	330	67	5.99%	68.85%
Other cardiomyopathy	6	57	51	4.56%	73.41%
Other lower respiratory infections*	8	52	44	3.95%	77.36%
Non-Hodgkin lymphoma	4	39	35	3.11%	80.47%
Chronic kidney disease due to hypertension	80	109	29	2.57%	83.04%
Chronic kidney disease due to diabetes mellitus type 2	9	33	24	2.12%	85.17%
Influenza*	4	17	13	1.13%	86.30%
Subarachnoid haemorrhage	20	31	11	0.99%	87.29%
Chronic kidney disease due to diabetes mellitus type 1	2	13	11	0.94%	88.23%
Non-rheumatic calcific aortic valve disease	35	45	10	0.87%	89.10%
Pedestrian road injuries	7	16	9	0.77%	89.87%

* These are a further subdivision of Level 4 causes, used for redistribution. In particular: “chronic ischemic heart disease” and “acute myocardial infarction” merge into Level 4 “ischemic heart disease”; “influenza”, “pneumococcal pneumonia” and “other lower respiratory infections” merge into Level 4 “lower respiratory infections”.

Supplementary Table 8s. Calabria 2017, deaths added during redistribution to the 15 most affected Level 4 underlying causes of death (overall deaths redistributed=4,303)

**Level 4 underlying cause of death**	**Deaths before redistribution**	**Deaths after redistribution**	**Deaths added**	**Percent of overall redistributed**	**Cumulative percentage**
Ischemic stroke	259	1536	1277	29.68%	29.68%
Diabetes mellitus type 2	76	860	783	18.20%	47.88%
Chronic ischemic heart disease*	1469	2015	546	12.69%	60.57%
Intracerebral haemorrhage	298	607	310	7.19%	67.76%
Acute myocardial infarction*	737	1000	262	6.10%	73.86%
Other cardiomyopathy	9	160	150	3.49%	77.35%
Chronic kidney disease due to hypertension	245	359	114	2.65%	80.00%
Non-Hodgkin lymphoma	25	135	110	2.55%	82.54%
Chronic kidney disease due to diabetes mellitus type 2	23	111	88	2.05%	84.59%
Other lower respiratory infections*	17	102	85	1.98%	86.57%
Influenza*	20	85	65	1.51%	88.08%
Subarachnoid haemorrhage	60	113	53	1.23%	89.31%
Non-rheumatic calcific aortic valve disease	78	118	39	0.91%	90.23%
Pedestrian road injuries	26	64	39	0.90%	91.13%
Motor vehicle road injuries	40	73	33	0.77%	91.89%

* These are a further subdivision of Level 4 causes, used for redistribution. In particular: “chronic ischemic heart disease” and “acute myocardial infarction” merge into Level 4 “ischemic heart disease”; “influenza”, “pneumococcal pneumonia” and “other lower respiratory infections” merge into Level 4 “lower respiratory infections”.

Supplementary Table 8t. Sardegna 2017, deaths added during redistribution to the 15 most affected Level 4 underlying causes of death (overall deaths redistributed=2,745)

Level 4 underlying cause of death	Deaths before redistribution	Deaths after redistribution	Deaths added	Percent of overall redistributed	Cumulative percentage
Ischemic stroke	274	981	707	25.74%	25.74%
Diabetes mellitus type 2	85	416	331	12.07%	37.81%
Chronic ischemic heart disease*	782	1071	290	10.55%	48.36%
Acute myocardial infarction*	605	798	194	7.05%	55.41%
Intracerebral haemorrhage	228	419	190	6.94%	62.35%
Non-Hodgkin lymphoma	13	148	135	4.90%	67.25%
Influenza*	31	150	119	4.32%	71.58%
Other lower respiratory infections*	18	127	109	3.97%	75.55%
Chronic kidney disease due to hypertension	181	271	90	3.26%	78.81%
Other cardiomyopathy	14	85	71	2.59%	81.41%
Chronic kidney disease due to diabetes mellitus type 2	32	95	64	2.32%	83.73%
Pneumococcal pneumonia*	2	47	45	1.65%	85.38%
Non-rheumatic calcific aortic valve disease	139	177	38	1.39%	86.77%
Subarachnoid haemorrhage	35	66	31	1.12%	87.89%
Pedestrian road injuries	19	43	24	0.88%	88.77%

* These are a further subdivision of Level 4 causes, used for redistribution. In particular: “chronic ischemic heart disease” and “acute myocardial infarction” merge into Level 4 “ischemic heart disease”; “influenza”, “pneumococcal pneumonia” and “other lower respiratory infections” merge into Level 4 “lower respiratory infections”.

Supplementary Table 8u. Sicilia 2017, deaths added during redistribution to the 15 most affected Level 4 underlying causes of death (overall deaths redistributed=11,956)

Level 4 underlying cause of death	Deaths before redistribution	Deaths after redistribution	Deaths added	Percent of overall redistributed	Cumulative percentage
Ischemic stroke	822	4775	3953	33.06%	33.06%
Diabetes mellitus type 2	334	2350	2016	16.86%	49.93%
Chronic ischemic heart disease*	3144	4373	1229	10.28%	60.21%
Intracerebral haemorrhage	732	1651	919	7.69%	67.89%
Acute myocardial infarction*	1987	2687	701	5.86%	73.75%
Other lower respiratory infections*	57	434	377	3.15%	76.90%
Other cardiomyopathy	21	390	369	3.09%	79.99%
Non-Hodgkin lymphoma	42	370	328	2.74%	82.73%
Chronic kidney disease due to hypertension	718	1020	302	2.53%	85.26%
Chronic kidney disease due to diabetes mellitus type 2	52	315	263	2.20%	87.46%
Influenza*	30	271	241	2.02%	89.48%
Subarachnoid haemorrhage	117	270	153	1.28%	90.76%
Non-rheumatic calcific aortic valve disease	209	307	98	0.82%	91.58%
Pedestrian road injuries	50	133	83	0.69%	92.27%
Chronic kidney disease due to diabetes mellitus type 1	19	101	82	0.69%	92.96%

* These are a further subdivision of Level 4 causes, used for redistribution. In particular: “chronic ischemic heart disease” and “acute myocardial infarction” merge into Level 4 “ischemic heart disease”; “influenza”, “pneumococcal pneumonia” and “other lower respiratory infections” merge into Level 4 “lower respiratory infections”.

Supplementary Table 9. Italy, year 2017, top 15 Garbage Code packages, by number of deaths, and redistribution proportions to Underlying Causes of Death.

Ill-defined package	Total package attributed deaths	Underlying cause of death	Number of deaths	Cause percentage	cumulative
Unspecified type of Stroke	33418	Ischemic stroke	27467	82.19%	82.19%
		Intracerebral hemorrhage	5063	15.15%	97.34%
		Subarachnoid hemorrhage	887	2.66%	100.00%
Diabetes unspecified type	14692	Diabetes mellitus type 2	14592	99.32%	99.32%
		Diabetes mellitus type 1	100	0.68%	100.00%
Unspecified Heart Diseases	11313	Chronic ischemic heart disease*	4617	40.81%	40.81%
		Hypertensive heart disease	2798	24.73%	65.54%
		Acute myocardial infarction*	2381	21.04%	86.59%
		Non-rheumatic calcific aortic valve disease	450	3.98%	90.56%
		Rheumatic heart disease	245	2.16%	92.72%
		Endocarditis	207	1.83%	94.55%
		Non-rheumatic degenerative mitral valve disease	106	0.94%	95.49%
		Other cardiomyopathy	84	0.75%	96.24%
		Myocarditis	47	0.42%	96.66%
		Other cardiovascular and circulatory diseases (internal)	35	0.31%	96.96%
Unspecified lower respiratory infectious	10955	Influenza*	4470	40.81%	40.81%
		Pneumococcal pneumonia*	3641	33.24%	74.05%
		Other lower respiratory infections*	2701	24.66%	98.71%
		H influenzae type B pneumonia	72	0.65%	99.36%
		Respiratory syncytial virus pneumonia	70	0.64%	100.00%
Exposure to unspecified factor X59	8728	Falls	7016	80.39%	80.39%
		Pedestrian road injuries	534	6.12%	86.51%
		Motor vehicle road injuries	370	4.24%	90.75%
		Pulmonary aspiration and foreign body in airway	165	1.89%	92.64%
		Other exposure to mechanical forces	139	1.59%	94.23%
		Adverse effects of medical treatment	97	1.11%	95.35%
		Environmental heat and cold exposure	87	1.00%	96.35%
		Other unintentional injuries	81	0.93%	97.28%
		Motorcyclist road injuries	44	0.51%	97.78%
		Other transport injuries	40	0.45%	98.24%
Sepsis (Non- maternal and neonatal sepsis)	7637	Ischemic stroke	447	5.85%	5.85%
		Colon and rectum cancer	369	4.83%	10.68%
		Chronic obstructive pulmonary disease	332	4.35%	15.03%
		Diabetes mellitus type 2	329	4.31%	19.34%
		Chronic kidney disease due to hypertension	292	3.82%	23.16%
		Other non-Hodgkin lymphoma	266	3.48%	26.64%
		Gallbladder and biliary diseases	263	3.44%	30.08%
		Urinary tract infections and interstitial nephritis	248	3.25%	33.33%
		Paralytic ileus and intestinal obstruction	236	3.08%	36.42%
		Other lower respiratory infections*	228	2.99%	39.40%

Ill-defined package	Total package attributed deaths	Primary cause of death	Number of deaths	Cause percentage	cumulative
Unspecified Cancer Site	6703	Tracheal, bronchus, and lung cancer	1172	17.49%	17.49%
		Colon and rectum cancer	931	13.88%	31.38%
		Prostate cancer	860	12.83%	44.21%
		Breast cancer	746	11.13%	55.34%
		Stomach cancer	486	7.26%	62.59%
		Pancreatic cancer	422	6.30%	68.90%
		Bladder cancer	259	3.86%	72.75%
		Other non-Hodgkin lymphoma	247	3.69%	76.44%
		Other malignant neoplasms (internal)	245	3.66%	80.11%
		Kidney cancer	144	2.14%	82.25%
Shock, Cardiac Attest, Coma	5551	Chronic ischemic heart disease*	710	12.79%	12.79%
		Acute myocardial infarction*	499	8.99%	21.78%
		Tracheal, bronchus, and lung cancer	399	7.18%	28.97%
		Ischemic stroke	282	5.09%	34.05%
		Chronic obstructive pulmonary disease	271	4.87%	38.93%
		Hypertensive heart disease	240	4.32%	43.24%
		Colon and rectum cancer	216	3.89%	47.13%
		Intracerebral hemorrhage	169	3.04%	50.18%
		Diabetes mellitus type 2	147	2.64%	52.82%
		Cirrhosis and other chronic liver diseases	142	2.56%	55.38%
Heart failure unspecified right or left	5457	Chronic ischemic heart disease*	1215	22.27%	22.27%
		Hypertensive heart disease	1027	18.82%	41.09%
		Acute myocardial infarction*	613	11.24%	52.33%
		Chronic obstructive pulmonary disease	344	6.30%	58.63%
		Ischemic stroke	271	4.96%	63.59%
		Chronic kidney disease due to hypertension	270	4.95%	68.54%
		Other cardiomyopathy	251	4.60%	73.14%
		Diabetes mellitus type 2	204	3.73%	76.87%
		Non-rheumatic calcific aortic valve disease	182	3.34%	80.21%
		Tracheal, bronchus, and lung cancer	130	2.38%	82.59%
Senility	5451	Chronic ischemic heart disease*	967	17.75%	17.75%
		Hypertensive heart disease	752	13.80%	31.55%
		Chronic obstructive pulmonary disease	479	8.79%	40.34%
		Acute myocardial infarction*	328	6.02%	46.36%
		Ischemic stroke	265	4.87%	51.23%
		Colon and rectum cancer	185	3.40%	54.62%
		Chronic kidney disease due to hypertension	170	3.12%	57.74%
		Tracheal, bronchus, and lung cancer	147	2.70%	60.44%
		Breast cancer	137	2.51%	62.95%
		Intracerebral hemorrhage	134	2.45%	65.40%

Ill-defined package	Total package attributed deaths	Underlying cause of death	Number of deaths	Cause percentage	cumulative
Hypertension	4709	Hypertensive heart disease	1086	23.07%	23.07%
		Intracerebral hemorrhage	940	19.96%	43.03%
		Chronic ischemic heart disease*	783	16.64%	59.67%
		Ischemic stroke	738	15.68%	75.35%
		Acute myocardial infarction*	513	10.90%	86.25%
		Chronic kidney disease due to hypertension	499	10.60%	96.85%
		Subarachnoid hemorrhage	80	1.71%	98.56%
		Aortic aneurysm	63	1.33%	99.89%
		Angina	5	0.11%	100.00%
		Ischemic heart disease	0	0.00%	100.00%
Other ill-defined causes of death	4639	Tracheal, bronchus, and lung cancer	296	6.38%	6.38%
		Chronic ischemic heart disease*	286	6.16%	12.76%
		Acute myocardial infarction*	208	4.47%	18.92%
		Chronic obstructive pulmonary disease	156	3.37%	23.39%
		Colon and rectum cancer	153	3.29%	26.76%
		Hypertensive heart disease	153	3.29%	30.06%
		Cirrhosis and other chronic liver diseases	100	2.15%	33.35%
		Pancreatic cancer	98	2.11%	35.50%
		Breast cancer	91	1.97%	37.61%
		Self-harm by other specified means	80	1.72%	39.58%
Non-follicular lymphoma, unspecified	3978	Other non-Hodgkin lymphoma	3836	96.42%	96.42%
		Burkitt lymphoma	142	3.58%	100.00%
Primary or secondary Liver Cancer Unspecified	3850	Colon and rectum cancer	867	22.53%	22.53%
		Pancreatic cancer	523	13.57%	36.10%
		Stomach cancer	458	11.90%	48.00%
		Prostate cancer	413	10.73%	58.73%
		Bladder cancer	350	9.10%	67.83%
		Breast cancer	341	8.87%	76.70%
		Liver cancer	265	6.88%	83.58%
		Kidney cancer	198	5.13%	88.71%
		Gallbladder and biliary tract cancer	145	3.78%	92.49%
		Esophageal cancer	105	2.73%	95.22%
Unspecified cardiovascular diseases	3702	Chronic ischemic heart disease*	1150	31.07%	31.07%
		Hypertensive heart disease	697	18.82%	49.89%
		Acute myocardial infarction*	615	16.62%	66.51%
		Intracerebral hemorrhage	243	6.56%	73.07%
		Ischemic stroke	241	6.51%	79.58%
		Chronic kidney disease due to hypertension	169	4.57%	84.15%
		Non-rheumatic calcific aortic valve disease	109	2.94%	87.09%
		Aortic aneurysm	79	2.14%	89.23%
		Rheumatic heart disease	61	1.65%	90.89%
		Lower extremity peripheral arterial disease	53	1.43%	92.32%

* These causes are a further subdivision of Level 4 causes used for redistribution. In particular: “chronic ischemic heart disease” and “acute myocardial infarction” merge into Level 4 “ischemic heart disease”; “influenza”, “pneumococcal pneumonia” and “other lower respiratory infections” merge into Level 4 “lower respiratory infections”.

Supplementary Table 10. Italy, year 2017, top 15 underlying causes of death by number of redistributed deaths, and main Garbage Code packages from which these are originating.

Cause Name	Overall number of redistributed deaths to Underlying CoD	Package Description	Deaths redistributed to Underlying CoD	Package percentage	Cumulative percentage
Ischemic Stroke	32603	Unspecified type of Stroke	27465	84.24%	84.24%
		Hypertension	738	2.26%	86.50%
		Cardiac rhythm disorders	465	1.43%	87.93%
		Pneumonitis	450	1.38%	89.31%
		Sepsis (Non- maternal and neonatal sepsis)	447	1.37%	90.68%
		Atherosclerosis	389	1.19%	91.87%
		Shock, Cardiac Arrest, Coma	282	0.87%	92.74%
		Plegia	279	0.86%	93.60%
		Heart failure unspecified right or left	271	0.83%	94.43%
		Senility	265	0.81%	95.24%
Diabetes mellitus type 2	16109	Diabetes unspecified type	14592	90.58%	90.58%
		Sepsis (Non- maternal and neonatal sepsis)	329	2.05%	92.63%
		Heart failure unspecified right or left	204	1.26%	93.89%
		Acute kidney failure	196	1.22%	95.11%
		Fluid, Electrolyte, Acid Base Disorders	171	1.06%	96.17%
		Shock, Cardiac Arrest, Coma	147	0.91%	97.08%
		Left heart failure	143	0.89%	97.97%
		Osteomyelitis	69	0.43%	98.40%
		Senility	62	0.38%	98.79%
		Pneumonitis	34	0.21%	99.00%
Chronic ischemic heart disease*	13172	Unspecified Heart Diseases	4617	35.05%	35.05%
		Heart failure unspecified right or left	1215	9.22%	44.28%
		Unspecified cardiovascular diseases	1150	8.73%	53.01%
		Cardiac rhythm disorders	1098	8.34%	61.35%
		Senility	967	7.34%	68.69%
		Left heart failure	890	6.76%	75.45%
		Hypertension	783	5.95%	81.40%
		Shock, Cardiac Arrest, Coma	710	5.39%	86.79%
		Other ill-defined causes of death	286	2.17%	88.96%
		Atherosclerosis	270	2.05%	91.00%
Intracerebral hemorrhage	7625	Unspecified type of Stroke	5062	66.39%	66.39%
		Hypertension	940	12.33%	78.72%
		Unspecified cardiovascular diseases	243	3.19%	81.91%
		Shock, Cardiac Arrest, Coma	169	2.21%	84.12%
		Intermediate cause for CNS	169	2.21%	86.34%
		Sepsis (Non- maternal and neonatal sepsis)	139	1.83%	88.17%
		Senility	134	1.75%	89.92%
		Cardiac rhythm disorders	119	1.55%	91.47%
		Chronic respiratory failure	97	1.27%	92.74%
		Plegia	83	1.09%	93.83%

Acute myocardial infraction*	7005	Unspecified Heart Diseases	2381	33.99%	33.99%
		Cardiac rhythm disorders	628	8.96%	42.95%
		Unspecified cardiovascular diseases	615	8.78%	51.73%
		Heart failure unspecified right or left	613	8.76%	60.49%
		Hypertension	513	7.33%	67.82%
		Shock, Cardiac Arrest, Coma	499	7.13%	74.95%
		Left heart failure	416	5.94%	80.89%
		Senility	328	4.68%	85.57%
		Other ill-defined causes of death	208	2.96%	88.54%
		Acute kidney failure	124	1.77%	90.30%
Influenza*	5100	Unspecified lower respiratory infections	4470	87.66%	87.66%
		Sepsis (Non- maternal and neonatal sepsis)	156	3.06%	90.71%
		Pleurisy, Pyothorax	114	2.24%	92.95%
		Acute kidney failure	90	1.76%	94.71%
		Chronic respiratory failure	72	1.40%	96.11%
		Shock, Cardiac Arrest, Coma	41	0.81%	96.92%
		Fluid, Electrolyte, Acid Base Disorders	36	0.70%	97.62%
		Acute Respiratory Failure	28	0.54%	98.17%
		Senility	20	0.38%	98.55%
		Pneumothorax	18	0.34%	98.90%
Other non-Hodgkin lymphoma	4789	Non-follicular lymphoma, unspecified	3836	80.11%	80.11%
		Sepsis (Non- maternal and neonatal sepsis)	266	5.55%	85.65%
		Unspecified Cancer Site	247	5.17%	90.82%
		Pleurisy, Pyothorax	83	1.73%	92.54%
		Gastrointestinal Bleeding	71	1.48%	94.02%
		Abdomen and Pelvis Cancer	65	1.35%	95.37%
		Shock, Cardiac Arrest, Coma	61	1.27%	96.64%
		Acute kidney failure	53	1.10%	97.74%
		Pulmonary Embolism	17	0.36%	98.10%
		Acute Respiratory Failure	16	0.33%	98.43%
Other cardiomyopathy	4235	Unspecified Cardiomyopathy	2917	68.89%	68.89%
		Myocardial Degeneration	517	12.21%	81.10%
		Heart failure unspecified right or left	251	5.92%	87.02%
		Left heart failure	133	3.14%	90.16%
		Amyloidosis	108	2.56%	92.72%
		Cardiac rhythm disorders	86	2.04%	94.76%
		Unspecified Heart Diseases	84	1.99%	96.75%
		Shock, Cardiac Arrest, Coma	57	1.34%	98.09%
		Pulmonary Embolism	22	0.51%	98.60%
		Unspecified cardiovascular diseases	17	0.41%	99.01%

Pneumococcal pneumonia*	3924	Unspecified lower respiratory infections	3641	92.80%	92.80%
		Pleurisy, Pyothorax	58	1.48%	94.28%
		Acute kidney failure	47	1.19%	95.47%
		Chronic respiratory failure	39	1.00%	96.46%
		Sepsis (Non- maternal and neonatal sepsis)	39	0.99%	97.45%
		Shock, Cardiac Arrest, Coma	22	0.57%	98.02%
		Fluid, Electrolyte, Acid Base Disorders	20	0.52%	98.54%
		Acute Respiratory Failure	15	0.39%	98.93%
		Pneumonitis	13	0.33%	99.27%
		Pneumothorax	10	0.26%	99.53%
Other lower respiratory infections*	3393	Unspecified lower respiratory infections	2701	79.61%	79.61%
		Sepsis (Non- maternal and neonatal sepsis)	228	6.73%	86.34%
		Acute kidney failure	79	2.32%	88.67%
		Pleurisy, Pyothorax	76	2.23%	90.89%
		Chronic respiratory failure	70	2.07%	92.96%
		Shock, Cardiac Arrest, Coma	51	1.52%	94.48%
		Senility	33	0.97%	95.45%
		Cerebral Palsy	31	0.90%	96.35%
		Fluid, Electrolyte, Acid Base Disorders	29	0.85%	97.21%
		Acute Respiratory Failure	26	0.77%	97.97%
Chronic kidney disease due to hypertension	3096	Anemia Unspecified	525	16.97%	16.97%
		Hypertension	499	16.12%	33.08%
		Sepsis (Non- maternal and neonatal sepsis)	292	9.42%	42.51%
		Heart failure unspecified right or left	270	8.72%	51.23%
		Acute kidney failure	257	8.31%	59.55%
		Left heart failure	194	6.26%	65.81%
		Senility	170	5.49%	71.30%
		Unspecified cardiovascular diseases	169	5.46%	76.76%
		Fluid, Electrolyte, Acid Base Disorders	127	4.11%	80.87%
		Shock, Cardiac Arrest, Coma	114	3.69%	84.56%
Chronic kidney disease due to diabetes mellitus type 2	2147	CKD due to diabetes Unspecified type	1669	77.71%	77.71%
		Acute kidney failure	74	3.44%	81.15%
		Heart failure unspecified right or left	67	3.12%	84.26%
		Anemia Unspecified	51	2.35%	86.62%
		Sepsis (Non- maternal and neonatal sepsis)	49	2.30%	88.92%
		Fluid, Electrolyte, Acid Base Disorders	44	2.03%	90.95%
		Pleurisy, Pyothorax	42	1.95%	92.90%
		Shock, Cardiac Arrest, Coma	35	1.64%	94.54%
		Left heart failure	25	1.18%	95.73%
		Cardiac rhythm disorders	25	1.17%	96.90%

Subarachnoid hemorrhage	1258	Unspecified type of Stroke	887	70.56%	70.56%
		Hypertension	80	6.39%	76.95%
		Intermediate cause for CNS	52	4.12%	81.07%
		Sepsis (Non- maternal and neonatal sepsis)	35	2.80%	83.87%
		Shock, Cardiac Arrest, Coma	34	2.74%	86.60%
		Unspecified cardiovascular diseases	30	2.38%	88.98%
		Cardiac rhythm disorders	20	1.57%	90.56%
		Senility	19	1.50%	92.05%
		Chronic respiratory failure	17	1.35%	93.40%
		Other ill-defined causes of death	16	1.29%	94.69%
Non-rheumatic calcific aortic valve disease	1237	Unspecified Heart Diseases	450	36.36%	36.36%
		Heart failure unspecified right or left	182	14.75%	51.11%
		Left heart failure	160	12.95%	64.06%
		Unspecified cardiovascular diseases	109	8.79%	72.85%
		Senility	80	6.44%	79.29%
		Cardiac rhythm disorders	55	4.48%	83.77%
		Shock, Cardiac Arrest, Coma	39	3.17%	86.93%
		Acute kidney failure	39	3.13%	90.07%
		Sepsis (Non- maternal and neonatal sepsis)	37	2.95%	93.02%
		Other ill-defined causes of death	26	2.14%	95.16%
Pedestrian road injuries	804	Exposure to unspecified factor X59	534	66.39%	66.39%
		Unspecified Road Injuries	125	15.52%	81.91%
		External Causes UDI, type unspecified	67	8.29%	90.20%
		Unspecified CNS sign and symptom	14	1.71%	91.92%
		Sepsis (Non- maternal and neonatal sepsis)	13	1.57%	93.49%
		Unspecified Transport Injuries	12	1.49%	94.98%
		Other ill-defined causes of death	10	1.25%	96.24%
		CNS Abscess	7	0.87%	97.10%
		Schizophrenia	5	0.61%	97.71%
		Shock, Cardiac Arrest, Coma	4	0.49%	98.20%

* These causes are a further subdivision of Level 4 causes used for redistribution. In particular: “chronic ischemic heart disease” and “acute myocardial infarction” merge into Level 4 “ischemic heart disease”; “influenza”, “pneumococcal pneumonia” and “other lower respiratory infections” merge into Level 4 “lower respiratory infections”.

Authors' Contributions

Providing data or critical feedback on data sources

Gianfranco Alicandro, Ettore Beghi, Matthew Cunningham, Giovanni Damiani, Lorenzo Monasta, Christopher J L Murray, Mohsen Naghavi, Maja Pasovic, and Luca Ronfani.

Developing methods or computational machinery

Gianfranco Alicandro, Benedetta Armocida, Matthew Cunningham, Carlo La Vecchia, Matilde Leonardi, Francesca Giulia Magnani, Lorenzo Monasta, Christopher J L Murray, Mohsen Naghavi, Maja Pasovic, and Francesco S Violante.

Providing critical feedback on methods or results

Ettore Beghi, Giulia Carreras, Alberico L. Catapano, Matthew Cunningham, Simona Giampaoli, Gaetano Isola, Christopher J L Murray, Mohsen Naghavi, and Jorge Hugo Villafañe.

Drafting the work or revising is critically for important intellectual content

Luciana Albano, Gianfranco Alicandro, Benedetta Armocida, Massimiliano Beghi, Cristina Bosetti, Nicola Luigi Bragazzi, Giulio Castelpietra, Alberico L. Catapano, Maria Sofia Cattaruzza, Giulia Collatuzzo, Sara Conti, Giovanni Damiani, Pietro Ferrara, Carla Fornari, Silvano Gallus, Davide Golinelli, Gaetano Isola, Carlo La Vecchia, Paolo Lauriola, Matilde Leonardi, Francesca Giulia Magnani, Giada Minelli, Marcello Moccia, Lorenzo Monasta, Christopher J L Murray, Mohsen Naghavi, Maja Pasovic, Paolo Pedersini, Norberto Perico, Alberto Raggi, Giuseppe Remuzzi, Luca Ronfani, Francesco Sanmarchi, Davide Sattin, Brigid Unim, and Francesco S Violante.

Managing the overall research enterprise

Christopher J L Murray and Mohsen Naghavi.
