# Supplementary material for: Redistribution of garbage codes to underlying causes of death: a systematic analysis on Italy and a comparison with most populous Western European countries based on the Global Burden of Disease Study 2019
Source: Eur J Public Health. 2022 Jan 21;32(3):456–62. doi: 10.1093/eurpub/ckab194 (PMC9159332; doi:10.1093/eurpub/ckab194)
Supplement: ckab194_Supplementary_Data [file ckab194_supplementary_data.zip › PubMed_Italy_GC.docx]

GBD 2019 Italy Garbage Codes Collaborators

| **First Names** | **Last Name** |
| --- | --- |
| Lorenzo | Monasta |
| Gianfranco | Alicandro |
| Maja | Pasovic |
| Matthew | Cunningham |
| Benedetta | Armocida |
| Luciana | Albano |
| Ettore | Beghi |
| Massimiliano | Beghi |
| Cristina | Bosetti |
| Nicola Luigi | Bragazzi |
| Giulia | Carreras |
| Giulio | Castelpietra |
| Alberico L. | Catapano |
| Maria Sofia | Cattaruzza |
| Giulia | Collatuzzo |
| Sara | Conti |
| Giovanni | Damiani |
| Pietro | Ferrara |
| Carla | Fornari |
| Silvano | Gallus |
| Simona | Giampaoli |
| Davide | Golinelli |
| Gaetano | Isola |
| Paolo | Lauriola |
| Carlo | La Vecchia |
| Matilde | Leonardi |
| Francesca Giulia | Magnani |
| Giada | Minelli |
| Marcello | Moccia |
| Paolo | Pedersini |
| Norberto | Perico |
| Alberto | Raggi |
| Giuseppe | Remuzzi |
| Francesco | Sanmarchi |
| Davide | Sattin |
| Brigid | Unim |
| Jorge Hugo | Villafañe |
| Francesco S | Violante |
| Christopher J L | Murray |
| Luca | Ronfani |
| Mohsen | Naghavi |
